# Impact of bathymetry on Indian Ocean circulation in a nested regional ocean model

**DOI:** 10.1038/s41598-024-58464-2

**Published:** 2024-04-05

**Authors:** Raheema Rahman, Hasibur Rahaman

**Affiliations:** 1 Indian National Centre for Ocean Information Services (INCOIS), Ministry of Earth Sciences (MoES), Government of India, Hyderabad, 500090 India; 2 KUFOS-INCOIS Joint Research Centre, Kerala University of Fisheries and Ocean Studies, Panangad, Ernakulam, Kerala 682506 India

**Keywords:** Ocean model, Bathymetry, Boundary current, Deep circulation, Indian Ocean, Bay of Bengal, Climate sciences, Ocean sciences

## Abstract

The Regional Indian Ocean model based on Modular Ocean Model (MOM4p1) was used to understand the importance of a realistic representation of bathymetry on Ocean General Circulation. The model has 1/4° uniform horizontal resolution and is forced with Coordinated Ocean-Ice Reference Experiments (CORE-II) inter-annual forcing with two simulations named BLND (realistic bathymetry) and OM3 (smoothed bathymetry), which only differ in the representation of bathymetry for the years 1992–2005. We also used recent reanalysis products from ORAS5 and SODA3 and ADCP observation to compare the subsurface currents. We show that by the inclusion of realistic bathymetry, there is a significant improvement in the upper ocean salinity, temperature, and currents, particularly near the coast. The salinity and temperature of the upper ocean are very close to the observed value near the coast. The bias in the salinity and temperature was reduced to half in BLND simulation compared to OM3, which led to a more realistic East India Coastal Current (EICC). We show the first evidence of a basin-wide cyclonic gyre over the Bay of Bengal at 1000 m depth during spring, which is just opposite to that of a basin-wide anti-cyclonic gyre at the surface. We found the presence of poleward EICC during spring at 1000 m and 2000 m depth, which is opposite to that of the surface. The presence of this deeper EICC structure is completely absent during fall. We show the presence of a boundary current along the coast of Andaman and Nicobar Island at a depth of 2000 m. The observed Wyrtki Jet (WJ) magnitude and spatial structure are most realistically reproduced in BLND simulation as compared to OM3 simulations. Both ORAS5 and SODA reanalysis products underestimate the WJ magnitude. The presence of the Maldives Islands is responsible for the westward extent of Equatorial Under Current (EUC). The presence of Maldives also creates wakes on the leeward side in the EUC zonal current. During fall, EUC is better defined in the eastern Equatorial Indian Ocean and lies at a depth of between 50 and 100 m, unlike its spring counterpart, in which its core is located slightly deeper, between 100 and 150 m depth. During peak summer months, June–July, a strong eastward zonal jet is present at 1000 m depth, similar to Wyrtki Jet (WJ). Inter-monsoon Jets, i.e., spring and fall jets, are also seen but are in the opposite direction, i.e., westward, unlike eastward in WJ.

## Introduction

Despite being the third largest ocean in the world, the Indian Ocean (IO) remains the least sampled in space and time as compared to the Pacific and Atlantic Oceans. Hence, it remains the least well-understood open ocean basin in the world^1^. The primary driver for the IO circulation is the monsoon winds, which reverse the direction seasonally^2,3^. The variability of monsoon in the IO is thought to have an impact on mixed layer depth^4^ and the ocean-atmosphere interactions^5–9^. The seasonal reversal of the prominent upper ocean feature of the North IO circulation can be attributed to the reversing seasonal winds^10–12^. The other circulation features that change with season are the coastal currents along the coast of India and Somalia. During the summer (winter) monsoon, the West India Coastal Current (WICC) flows equatorward (poleward), and East India Coastal Current (EICC) flows poleward (equatorward)^13–17^. On the other hand, the Indian Ocean Dipole or IO Zonal Mode affects the inter-annual rainfall variation in the Indian and African subcontinent and the IO rim countries^8,9,18,19^. The IO climate anomalies affect other parts of the globe as well^8,12,20–23^. However, climate variability prediction for the IO is still at the experimental stage^12^.

For the planning of maritime activities, it is essential to accurately forecast specific oceanographic parameters such as currents, temperature, and salinity of surface and subsurface on different time scales^24^. Schott et al.^12^ have pointed out that, for better prediction, the essential requirements are enhanced observations and improved models. In recent times, through the IndOOS program, IO observing systems have now been reasonably well represented on a space-time scale^25^; however, models are still unable to simulate its mean and variability accurately^26^. Ocean model simulation errors are mainly caused by errors in forcing fields, model physics, and the representation of bathymetry. With the aim of improving the models, this study focuses on the importance of realistic representation of bathymetry in circulation models.

The ocean bathymetry can affect the ocean circulation by steering it and converting the energy of horizontal flow into vertically propagating waves. The ocean circulations are sensitive to large-scale bathymetric accuracy and resolution. Steering and mixing are the two important ways in which bathymetry influences circulation^27^. Ganachaud and Wunsch^28^ observed the enhanced mixing in the interior ocean with rough topography. The flat bottom calculation deficiencies in horizontal flow and stratification are rectified by the addition of bottom topography, which strengthened the boundary currents and diffusion in the experiment conducted by Spall and Pickart^29^. A similar effect of topography on subpolar gyre circulation is found by Winton^30^. From their study, Shetye et al.^16^ assume the possibility of error arising in the circulation theory of the Bay of Bengal (BoB) while ignoring the bottom topography. The Improvements in the regional model simulation with realistic topography and seasonal river runoff were studied by Rahaman et al.,^31^ who found that realistic topography is necessary to produce strong near-surface salinity gradients, resulting in realistic boundary currents simulation along the coast of India. From further study using CORE II simulations, Rahaman et al.^26^ states the necessity of improving the model physics and realistic representation of bathymetry rather than just enhancing the model’s horizontal resolution. However, those studies did not focus on equatorial and sub-surface currents. Hence, this study is focused on the impact of bathymetry on the IO sub-surface circulations, including deep currents.

Several studies were carried out around the globe on the effect of different islands using circulation models. Vos et al.^32^ demonstrate that the Sri Lankan Dome does not form in the absence of Sri Lankan landmass through numerical simulation. Gopalakrishnan and Cornuelle^33^ investigated the effect on regional scale ocean circulation by Palau Island and found that by only changing the bathymetry at Palau, there is a change in solution everywhere in the model domain, which is attributed to the eddy interactions and instabilities. Kersalé et al.^34^ found the importance of topography along with wind and inflow current, with a focus on Hawaiian islands. The effect of the Galapagos Islands on the Pacific cold tongue was reported by Karnauskas et al.^35^ using fine-resolution models. Whereas, its influence on tropical temperatures, currents, and generation of tropical instability waves was explored by Eden and Timmermann^36^.

In very recent times, the United States Office of Naval Research Flow Encountering Abrupt Topography (FLEAT) program was aimed to study the effect on open ocean current systems by Island chains and submerged ridges^37^. As a part of this study, there were six research cruises around Palau. It was found that the friction arising when the flow encounters topography spins up energetic ocean eddies. By linking the ocean basin scale with the topographic effect, the FLEAT program could progress in models and forecasts of relevant processes. Johnston et al.^37^ also discuss the importance of parameterizing the small-scale processes arising due to topography in relatively coarser basin and global scale models. To provide an understanding of the interactions between the flow and topography, there are numerous studies on the flow around headlands, over submarine ridges etc.^38–42^.

Signell and Geyer^39^ justifies the inclusion of horizontal eddy viscosity and no-slip condition in the model as a proxy for bottom friction in case the bathymetry is not accurately represented. Their study shows that the eddy viscosity plays a slight role unless coastal bathymetric variation is well represented. Farmer et al.^43^ state that when strong tides coincide with irregular coastlines, shear zones develop. Warner and MacCready^42^ demonstrated that parameterizing the drag resulting from rough topography improved the performance of hydrodynamic models. This finding aligns with the study conducted by Garett and Kunze^40^, which focused on the dependence of internal tide generation on topography. Magaldi et al.^41^ studied the lee wave generated around a topographic feature. Pattiaratchi et al.^44^ conclude that the vorticity balance is modified at regions with complex bottom topography, which makes it difficult to predict the eddy length scales in that area.

Chakraborty and Gangopadhyay^45^, in their study for the development of a high-resolution model for BoB, discuss the importance of bathymetry of the basin. Mukherjee et al.^46^ and Chatterjee et al.^47^ focused on the presence of Andaman and Nicobar Islands (ANI) in circulation models. The importance of accurately representing the ANI is implied in Chatterjee et al.^47^. Their results suggest that these islands influence the circulation in the Andaman Sea as well as the circulation of BoB even in the interior bay and along the coast of India. They observed that in the presence of ANI, a recirculation is happening within the Andaman Sea. The impact of the Island chain is not restricted to its neighborhood but extends to the East Coast of India, and it is important for modelling the circulation in BoB. Mukherjee et al.,^46^ using the Ocean General Circulation Model, studied the influence of ANI in creating eddies in western BoB (during the period 2011–2015). When the model was run without the ANI, the resultant eddies were notably less, compared to the model run which includes the ANI. However, in all these previous studies, the focus was mostly on surface circulation, but how the coastal and equatorial currents respond to the bathymetry change is missing. Also, the impact of bathymetry on deep circulation has not been reported yet. In this study, we showed these missing parts for the north IO.

Previous studies reported that the deficiency that arose in the model simulation of WICC might be due to the use of relatively smooth topography^31,48,49^. Karnauskas et al.^35^ found that using refined horizontal resolution along with incorporating the islands in the model led to a more realistic simulation of the Pacific cold tongue rather than introducing islands to a coarser model or refining the model without the islands. Even if the models simulate the seasonal cycle of currents, the intraseasonal variability is not properly captured by many in the coastal region, especially in the deeper depths^50^. Here in this study, we use identical models with refined resolution by only adjusting the bathymetry to study the impact of bathymetry alone in the IO region with special emphasis on the currents over the BoB and deeper currents in the Equatorial Indian Ocean (EIO). To the best of our knowledge, we have not found any such study that shows the impact of bathymetry on deeper currents in the IO. Hence, in this study, apart from surface circulation, we show the impact of bathymetry on deeper circulations as well.

The paper is organized as follows. Section “Model configuration” describes the model configuration, and section “Data used for model evaluation” gives the details of the data used for model evaluation. The results and discussion are included in section “Results and discussions”, which is organized as temperature in section “Temperature”, salinity in section “Salinity”, and currents in section “Currents”. Finally, summary and conclusion are given in section “Summary and conclusion”.

## Model configuration

This study employs the method of nesting a finer regional ocean model into a coarser global model by using Modular Ocean Model version 4p1^51^ (MOM4p1). The regional model domain covers the IO between 30° E–120 °E and 30 °S–30 °N. The global model configuration is analogous to the one used by Dunne et al.^52^ in the Earth system model. The IO is bounded by land on the northern and western sides, and an active boundary condition that allows mass transport is used in the eastern and southern boundaries of the regional model. The boundary conditions are taken from the global 1-degree model solution, which is also used in Rahaman et al.^31^.

**Figure 1:**
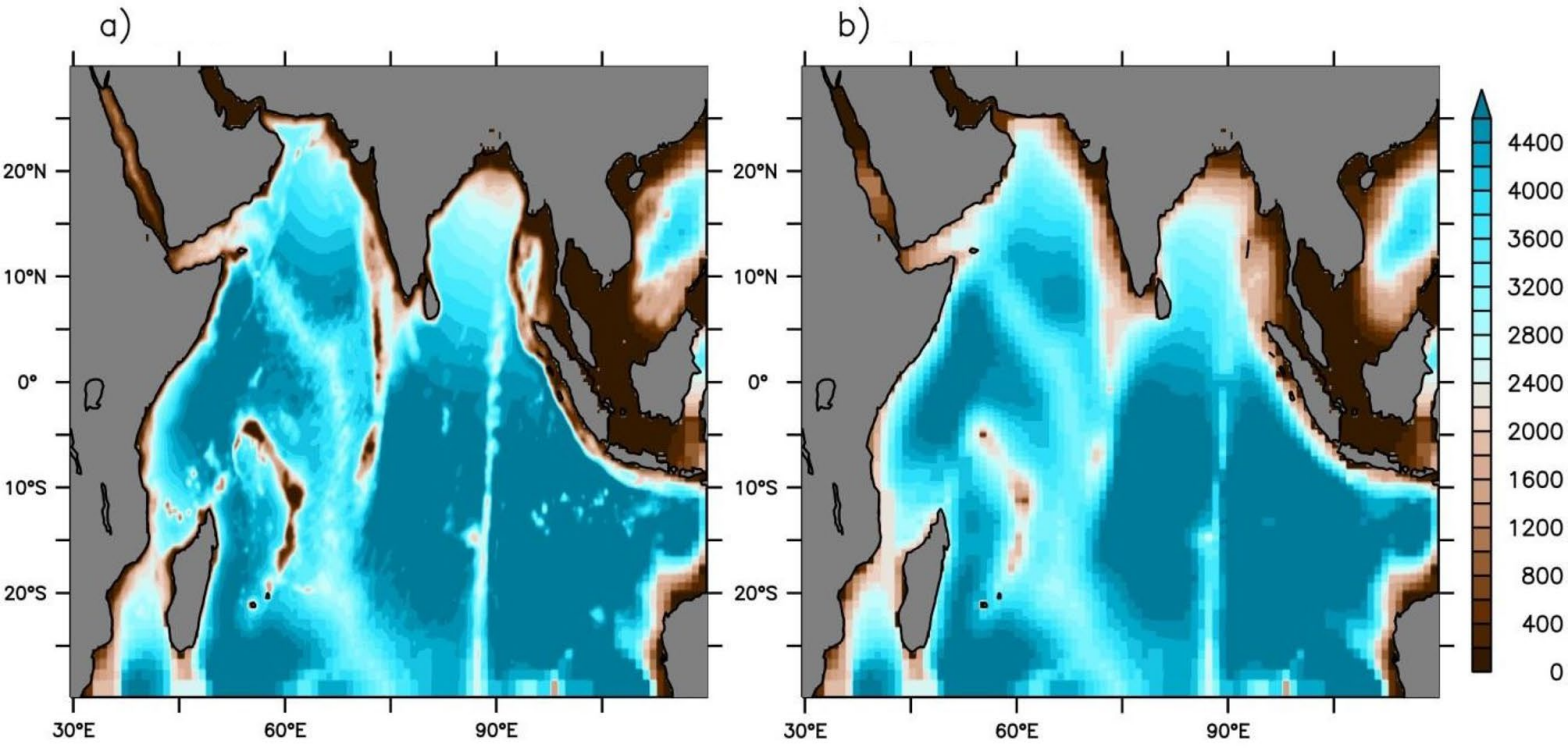
Bathymetry used in the regional models. The depth is given in meters. (**a**) Modified bathymetry (BLND), (**b**) smoothened bathymetry (OM3).

In the global model configuration, there are 50 z* vertical levels; among them, twenty-two (22) are spaced in the upper ocean (up to 220 m depth) with 10 m vertical resolution in the upper few levels. The remaining 28 vertical levels were distributed up to 5500 m in the global model. However, in the regional model, the vertical grid is further refined, having four vertical levels in the upper 5 m depth and then gradually increased to around 500 m to a bottom depth of 5.5 km. Details of model configurations and physics can be found in Rahaman et al.^31^ Two experimental runs are carried out and compared to study the influence of bathymetry in the Ocean General Circulation Model. The experiments are done by using two different bathymetry files used to configure the IO regional model. All the other factors of the model remain identical, i.e., boundary conditions, parametrization and mixing schemes, horizontal and vertical gridding, etc. We call the experiment with coarse bathymetry as OM3 and the one with refined bathymetry as BLND (Fig. 1). The BLND bathymetry is the improved bathymetric dataset by Sindhu et al.,^53^ which uses hydrographic data for the representation of shallower regions, while the OM3 uses the sea floor topography from Smith and Sandwell^54^. By comparing Fig. 1a and b, one can see that the variations at the coast, as well as the Island systems, are clearer and more realistic in the BLND bathymetry. Also, the forcing in these two experiments is the same, which is CORE-II inter-annual forcing^55^.

## Data used for model evaluation

We used several data to evaluate the model. To validate the temperature and salinity, we used Coriolis Ocean Re-Analysis (CORA) version 5 data. The CORA data is a global gridded data set generated from the in-situ temperature and salinity measurements from diverse global networks by Coriolis Data Centre^56^. Ocean Surface Currents Analyses Real-time (OSCAR) data is used to compare the surface currents averaged up to 30 m depth. This product is derived from satellite measurements^57^. The one-degree version was accessed from the APDRC site (http://apdrc.soest.hawaii.edu). The OSCAR product is validated against buoy measurements from EIO and Eastern BoB by Sikhakolli et al.^58^ and with ADCP by Mukherjee et al.^50^. Sikhakolli et al.^58^ showed that the Wyrtki jet appears during anomalous dipole years and is well captured in the OSCAR current. They also showed that OSCAR currents are in good agreement with buoy observed currents with a correlation exceeding 0.7. Mukherjee et al.^50^ in their study, showed that OSCAR current also matches well with ADCP observation. Hence, we used OSCAR gridded products to evaluate the spatial current simulations from OM3 and BLND.

For the subsurface currents, we used Masumoto et al.^59,60^ Acoustic Doppler Current Profiler (ADCP) data in the eastern IO. The vertical profile of the ADCP current data is analyzed from 40 to 200 m with the model output at 90° E equator. Reanalysis data are widely used as a proxy of observations for many research works. To see how the currents from reanalysis products perform for the subsurface current in the IO we used the two most widely used products, ORAS5 and SODA V3.3. The ORAS5 and SODA V3.3 were also obtained from the Asia Pacific Data Research Centre (APDRC). The Simple Ocean Data Assimilation (SODA) is a reliable university-based research product for seasonal climate time-scale ocean reanalysis. Version 3 of SODA uses MOM5/SIS at ¼ × ¼° for 50 vertical levels^61^. The Ocean Reanalysis System 5 (ORAS5) by ECMWF provides an estimate of the ocean state from 1979 to the present with a delay in a few days. The operational system of ECMWF to estimate the state of the global ocean is OCEAN5. It is a 5-ensemble member system generated using perturbed initial conditions assimilated with observation and forcing perturbation^62,63^. We used a 5-ensemble member average for our study.

## Results and discussions

### Temperature

**Figure 2:**
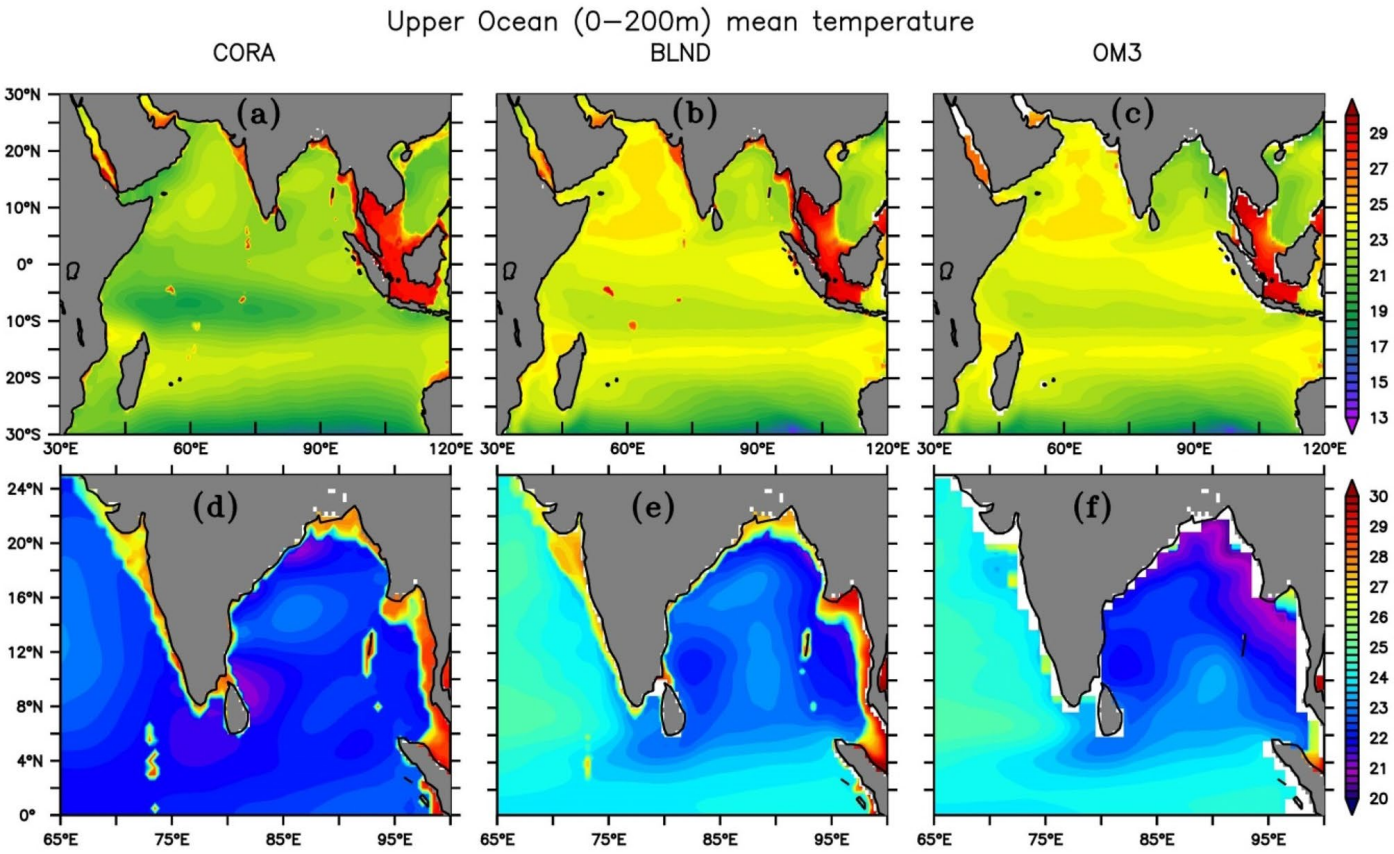
Mean temperature (in °C) from 1992 to 2005 for the upper ocean averaged over 0–200 m for (**a**, **d**) CORA, (**b**, **e**) BLND and (**c**, **f**) OM3. (**a**–**c**) Shows the Indian Ocean and (**d**–**f**) coast of India and along the Bay of Bengal.

Deep atmospheric convection in the tropical ocean is mainly driven by sea surface temperature (SST)^64–68^. Heat flux, advection, and mixing in the upper ocean determine the SST^69^. The warmest sea in the world ocean is the tropical north IO prior to the onset of summer monsoon, during April and May^70,71^. The physical properties of BoB show extreme variability, which makes it unique among other tropical ocean basins^2,17^. Shenoi et al.^71^ observed that the entire BoB is warmer throughout the year, with more warming in the northern BoB during summer with a temperature greater than 28 °C during August, which is favorable for active convection^65,66,72^. Despite improved model physics and resolution, models still lack accurate reproduction of the seasonal cycle of SST and the upper ocean temperature^26^. Figure 2 shows the spatial distribution of upper ocean (0–200 m) temperature from regional model simulations and CORA observation. Both simulations were able to capture the observed spatial mean patterns with basin-wide warmer upper ocean temperature as compared to CORA observation (Fig. 2a–c) except BoB, where OM3 shows cooler temperature as compared to CORA. The spatial distribution of BLND simulations remarkably matches that of CORA observation. Since the bathymetry changes are significant near the Indian coast (Fig. 1a,b), we show the upper ocean temperature for the eastern Arabian Sea and BoB (Fig. 2d–f). Although not much difference can be seen in BLND and OM3 simulations in the open ocean, significant differences are observed near the coast and ANI, where bathymetry shows large differences, particularly along the coast of India and eastern BoB. The bias (model-observation) in annual mean temperature is much less in BLND as compared to OM3 (smoothened bathymetry), which is evident in the northern and eastern boundaries of BoB and the west coast of India as well. Near the coast, BLND simulations almost reproduce the observed spatial pattern as well as the magnitude as compared to CORA observation. However, OM3 shows a much cooler temperature. The interior BoB also shows cooler upper ocean temperature in OM3 compared to BLND, which also corroborates the findings of Chatterjee et al.^47^. On the seasonal time scale, OM3 underestimates upper ocean temperature near the coast in the BoB during summer monsoon months. The temperature shows a cooler bias around the islands and near the coast. The OM3 simulation shows a negative (cool) bias greater than 5 °C in the north BoB very near the coast, whereas it is less than 4 °C in BLND (Figure not shown). A similar improvement in temperature of varied magnitude is observed in all the seasons with the use of realistic bathymetry in the model, i.e., in BLND simulation. The cool upper ocean temperature in OM3 simulations in the north BoB and very near the coast resulted due to the deeper bathymetry in the model (See Fig. 1). It can be seen from the figure that along the coast of India and Myanmar bathymetry shows a much deeper depth ~ 1000–2000 m in OM3 whereas in BLND its ranges 200–1000 m. Due to this deeper depth, the temperature near the coast and over northern BoB is cooler in the upper ocean (0–200 m) in OM3 simulation as compared to BLND simulations. This resulted from the shallower thermocline depth in OM3 simulation compared to BLND simulation, which is very close to the observed thermocline depth near the coasts and northern BoB (Figure not shown). Previous studies showed that observed diapycnal diffusivities over steep-sloping bottom topography^73^ and in tide-dominant marginal seas^74^ are 1–3 orders of magnitude higher than open ocean observed values ∼ 10^−5^ m^2^ s^−1^^75–77^. Simmons et al.,^78^ implemented the effects of this bottom-enhanced vertical mixing parameterization scheme in ocean general circulation models, which parameterizes diapycnal mixing over rough topography where stratification exists at depth. Additionally, the frictional dissipation of barotropic tidal energy and subsequent diapycnal tracer mixing is parameterized separately as in Lee et al.^79^. Since both simulations were performed identically, the improvements in temperature and salinity simulations in BLND compared to OM3 can also be attributed to the use of more realistic bathymetry and the implementation of effective tidal parametrization schemes^78,79^.

**Figure 3:**
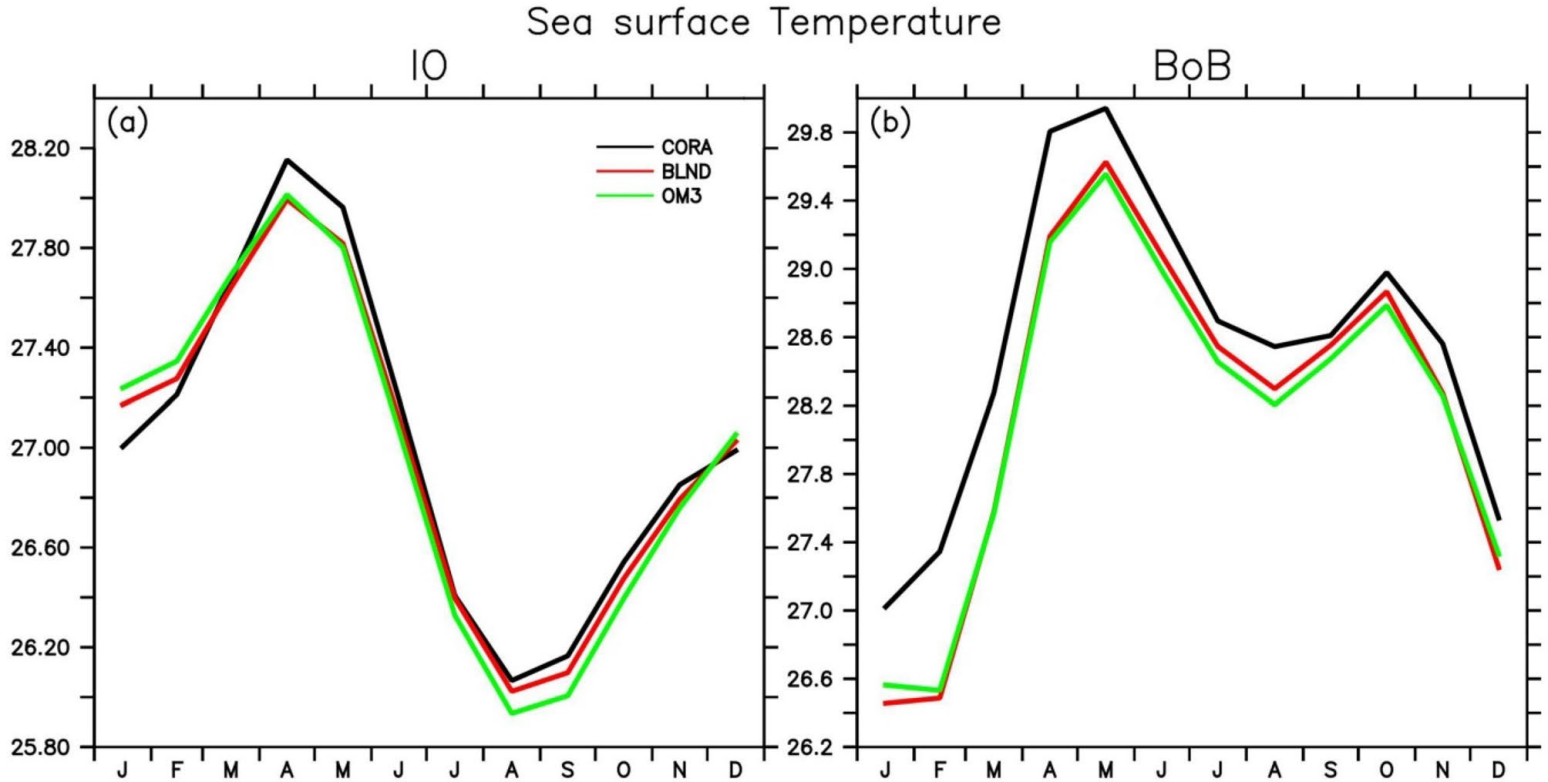
Seasonal cycle of sea surface temperature (in °C) averaged over (**a**) Indian Ocean (30° E–120° E, 30° S–30° N) and (**b**) Bay of Bengal (78° E–100° E, 6° N–25° N).

In order to see how the upper ocean temperature impacts SST, we show the seasonal cycle of SST averaged over the entire IO and BoB in Fig. 3. It can be seen that BLND solutions almost reproduced the CORA observed seasonal variation in the IO (Fig. 3a). OM3, too, shows SST very close to observations except in winter (spring) when it is slightly warmer (cooler) as compared to CORA observation. During summer, OM3 shows much cooler SST than CORA observation. However, this bias reduces in BLND and almost matches CORA values. In the BoB, both simulations show systematic cold bias with respect to CORA observation. However, BLND simulation performs better than OM3 (Fig. 3b). While analyzing the SST at a single point in Northern BoB (90° E, 20° N) (figure not included), it was observed that the OM3 simulations with smoothened bathymetry underestimate the CORA observation by more than 1 °C during January and February, which is around ~ 0.5 °C in the case with realistic bathymetry in BLND simulation. Bhat et al.^80^ emphasizes the importance of simulating the absolute value of SST for the convective activity. Hence, the realistic SST simulation of BLND simulation in the BoB will lead to a better understanding of the prediction of Indian Summer Monsoon Rainfall.

### Salinity

The thermodynamic structure of the upper ocean is influenced by surface buoyancy, which is mainly contributed by salinity^81,82^. The Arabian Sea and BoB receive varied amounts of rainfall, which leads to contrasting salinity distribution. The equatorial^83^ and north IO show an asymmetry in the salinity distribution; the Arabian Sea is saltier than BoB due to high evaporation^84^. Five of the largest rivers in the world join BoB north of 15° N. The surface layer of BoB is much fresher than that of the Arabian Sea. The salinity of BoB is highly heterogeneous^85^ with sharp fronts in all seasons, limiting the thickness of mixed layer depth in the BoB^71,86^. The stratification in the upper ocean in the north BoB is dominated by salinity rather than temperature^87^, owing to the large freshwater influx^88^ and precipitation exceeding evaporation^89^. In the case of BoB, the upper ocean is fresh compared to the other oceans^90^, which leads to a stable stratification^91,92^ and traps more heat which results in higher SST^71,93^. This freshwater has a substantial impact on air-sea interactions, thus affecting the climate of neighboring countries^94^. Hence, we evaluated the effect of bathymetry on upper ocean salinity simulations.

**Figure 4:**
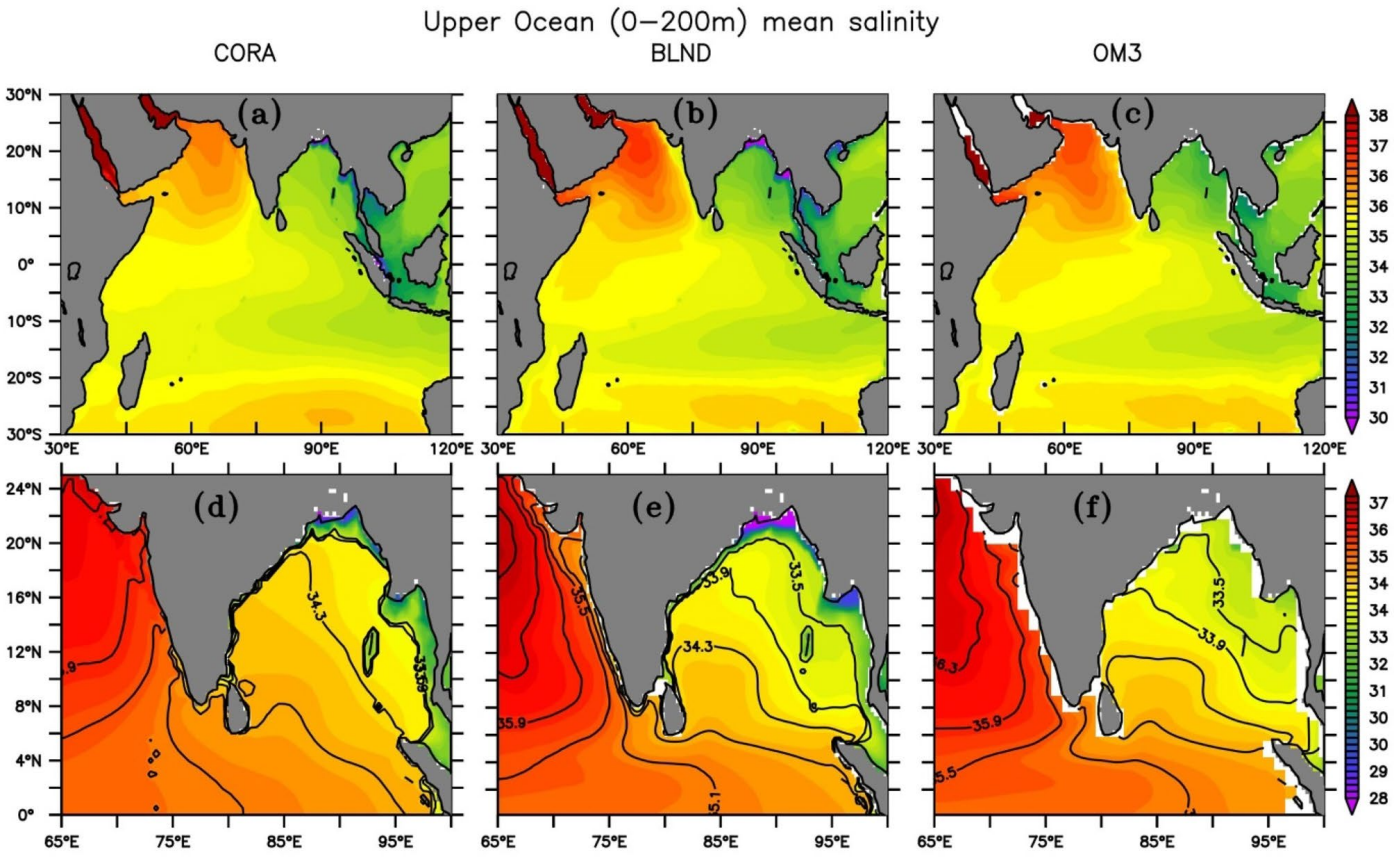
Same as Fig. 2 but for the salinity. The black lines in (**d**–**f**) show salinity contours.

Figure 4a–c(d–f) shows the upper ocean (0–200 m) mean salinity distribution over IO (BoB) from OM3, BLND simulations and CORA observations. BLND simulation matches the observed salinity distribution most realistically as compared to OM3 simulation. It can be seen that the salinity contours follow the bathymetry near the coast (Figs. 1a and 4e) in BLND simulation when compared with CORA observation. The CORA observation shows a contrasted pattern of salinity, with fresh waters of salinity less than 33 psu in the northeast portion of the bay and saltier water in the south and central basin. This structure is most realistic in BLND simulation as compared to OM3 simulation. This result also corroborates the findings of Benshila et al.^95^ Rahaman et al.^31^ found that the models with fine-scale topography exhibit strong near-surface stratification, which in turn affects the monsoon rainfall^96^. It is worth pointing out that both the temperature and salinity do not show any abrupt change near the southern boundary at 30° S or at the eastern boundary at 120° E. This is mainly due to the implementation of the most realistic lateral boundary condition in this nested regional model^31^. Recently Rahaman et al.^97^ showed the impact of the lateral boundary in the BoB circulation in the same model. Similar to the pattern observed in temperature, the salinity is also better simulated near the coast when realistic bathymetry is used instead of smoothened (Fig. 4d–f). The observed low salinity fronts along the coast of BoB are almost followed in the BLND simulation, whereas in the OM3 simulation, the low salinity fronts < 30 psu are absent. Benshila et al.^95^ also show reasonably good simulations made with NEMO models, but they showed it for only SSS. Here, we show the entire upper ocean (0–200 m), which is the most important part of the ocean for seasonal prediction^98^.

**Figure 5:**
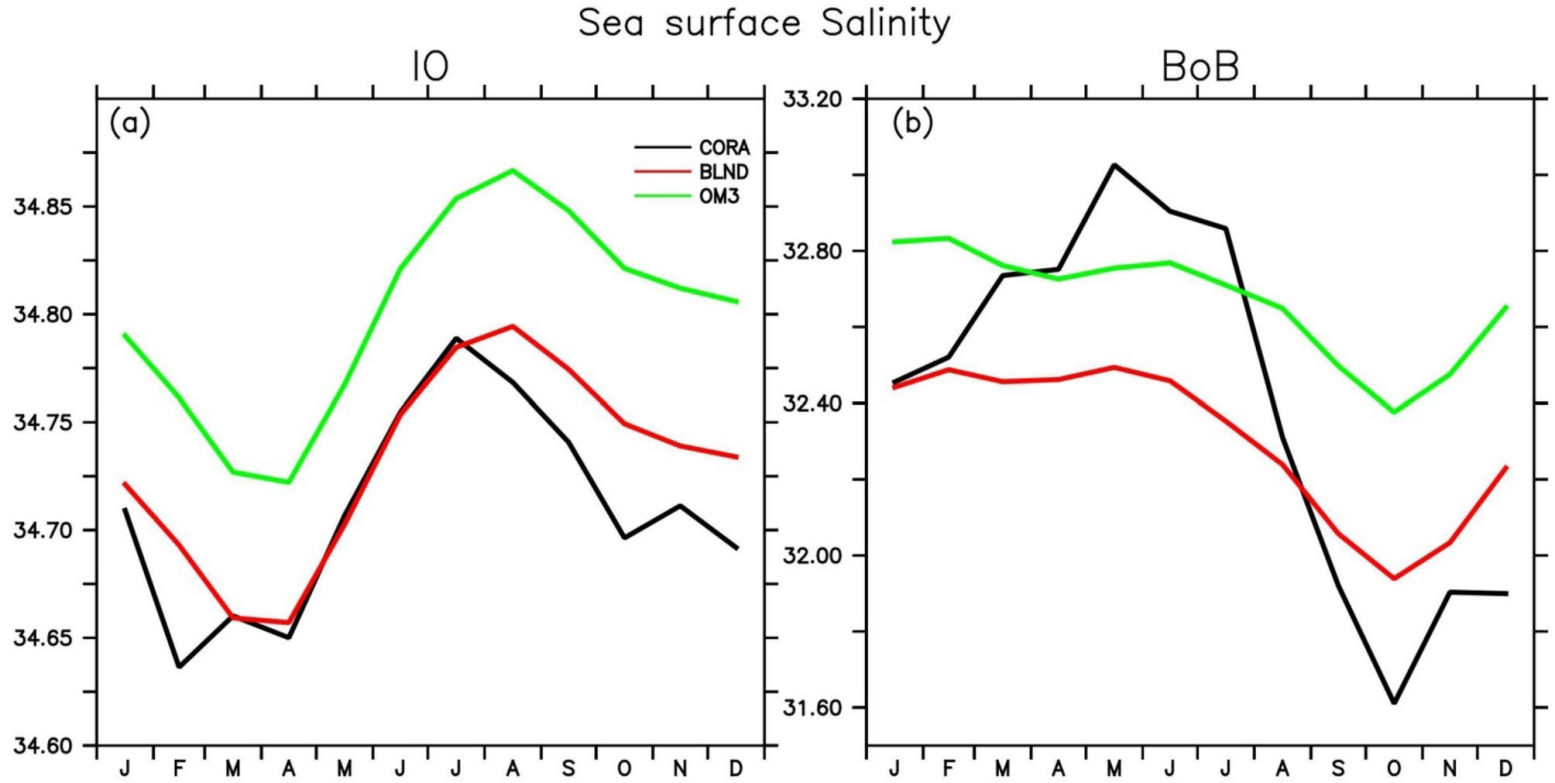
Same as Fig. 3 but for the Sea surface salinity.

**Figure 6:**
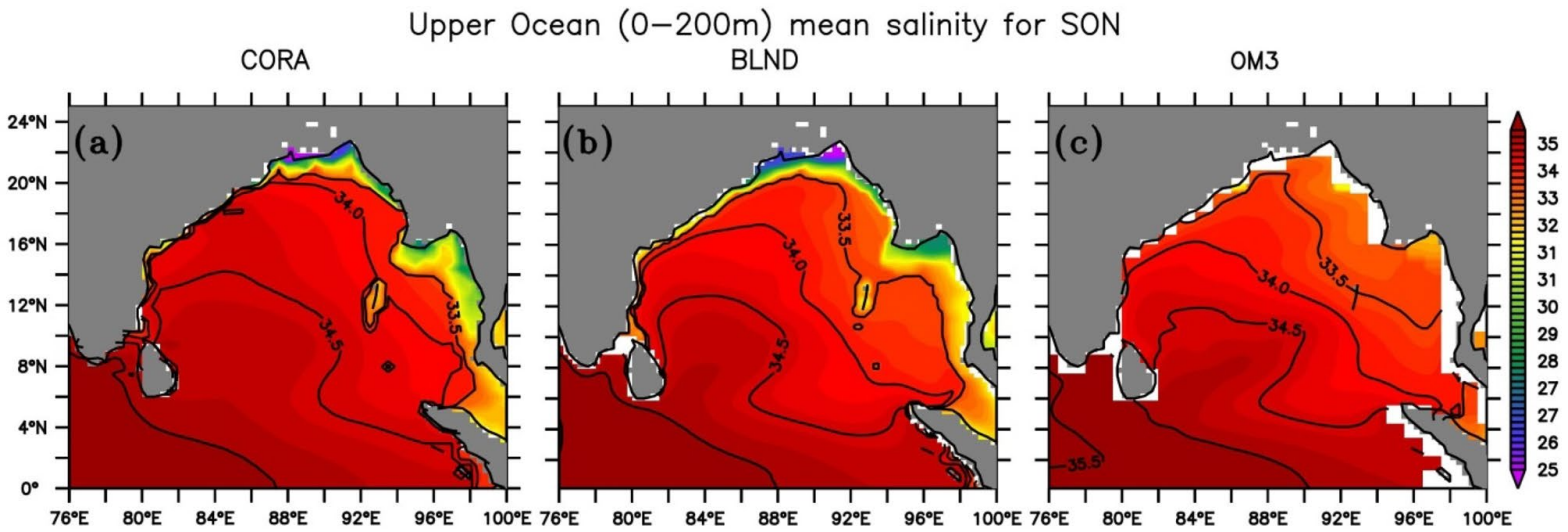
Mean salinity from 1992 to 2005 during fall (September–November) averaged over the surface to 200 m over the Bay of Bengal from (**a**) CORA, (**b**) BLND, and (**c**) OM3. The overlaid black lines are the salinity contours.

The seasonal variation of BLND sea surface salinity averaged over the entire IO basin (Fig. 5a) exactly follows that of the CORA observation from March to July, while OM3 overestimates the salinity for the entire year. The mismatch of BLND simulations after July could be due to the annual river runoff forcing in the model instead of seasonal or interannual river runoff forcing. In the BoB, BLND simulation is fresher than CORA observation during spring and summer, but it is slightly saltier for the rest of the year (Fig. 5b). The freshening of upper ocean salinity in October, as observed in CORA data after the peak monsoon is not captured by either of the simulations. This discrepancy may be attributed to the influence of annual river runoff forcing since the same model with seasonal river runoff forcing could capture this freshening in SSS (see Fig. 13 of Rahaman et al.^31^). Since BoB becomes most fresh after monsoon, to see how the model captures the spatial salinity pattern in the BoB, we show the upper ocean (0–200 m) salinity for September to November in Fig. 6. During the fall (SON), the salinity contours of BLND simulation follows that of CORA observation. A similar pattern of salinity is observed by Benshila et al.^95^ in agreement with Shankar et al.^17^. The salinity values less than 33 psu are observed in the CORA observation over the north BoB, which is very well captured in BLND simulations but absent in OM3 (Fig. 6c). The OM3 simulates salinity to a minimum of 33.5 psu, but in reality, the salinity values in the North BoB reach far below it, as synthesized by the BLND simulation^93,99^. The realistic salinity and temperature simulations in the BLND experiments compared to the OM3 experiment is mainly due to the fact that tidal parametrization schemes were more efficiently worked in realistic fine-scale bathymetry compared to coarse bathymetry in OM3^78^. Simulating the BoB salinity is challenging^87,88^. Overall, BLND simulation is very close to CORA observation as compared to OM3 simulation. This realistic salinity simulations in BLND simulation resulted due to realistic bathymetry and implementation of tidal parametrization schemes which works effectively over rough topography^78,79^.

### Currents

#### Surface current

Reversing monsoon winds are prominently observed to the north of 10° S in the IO^3,12^. The annual mean wind pattern is southwesterly as the summer monsoon winds are stronger than winter monsoon^71^ and have a wind pattern like that of July^100^. The mean current pattern over the IO shows the influence of the mean wind pattern and reverses its direction^3^. Jensen^101^ observed that during summer, the southwest monsoon current carries saltier Arabian Sea water to the BoB, which flows eastward along the south of Sri Lanka and turns to the north into BoB. Recently, Rahaman et al.^97^ have shown that this intrusion of saltier water from the Arabian Sea even occurs in the deeper layers between 300 and 1500 m depth at 8° N near the east coast of Sri Lanka. The northeast monsoon current flows westward across the basin during winter, which carries fresher BoB water to the Arabian Sea^12^.

**Figure 7:**
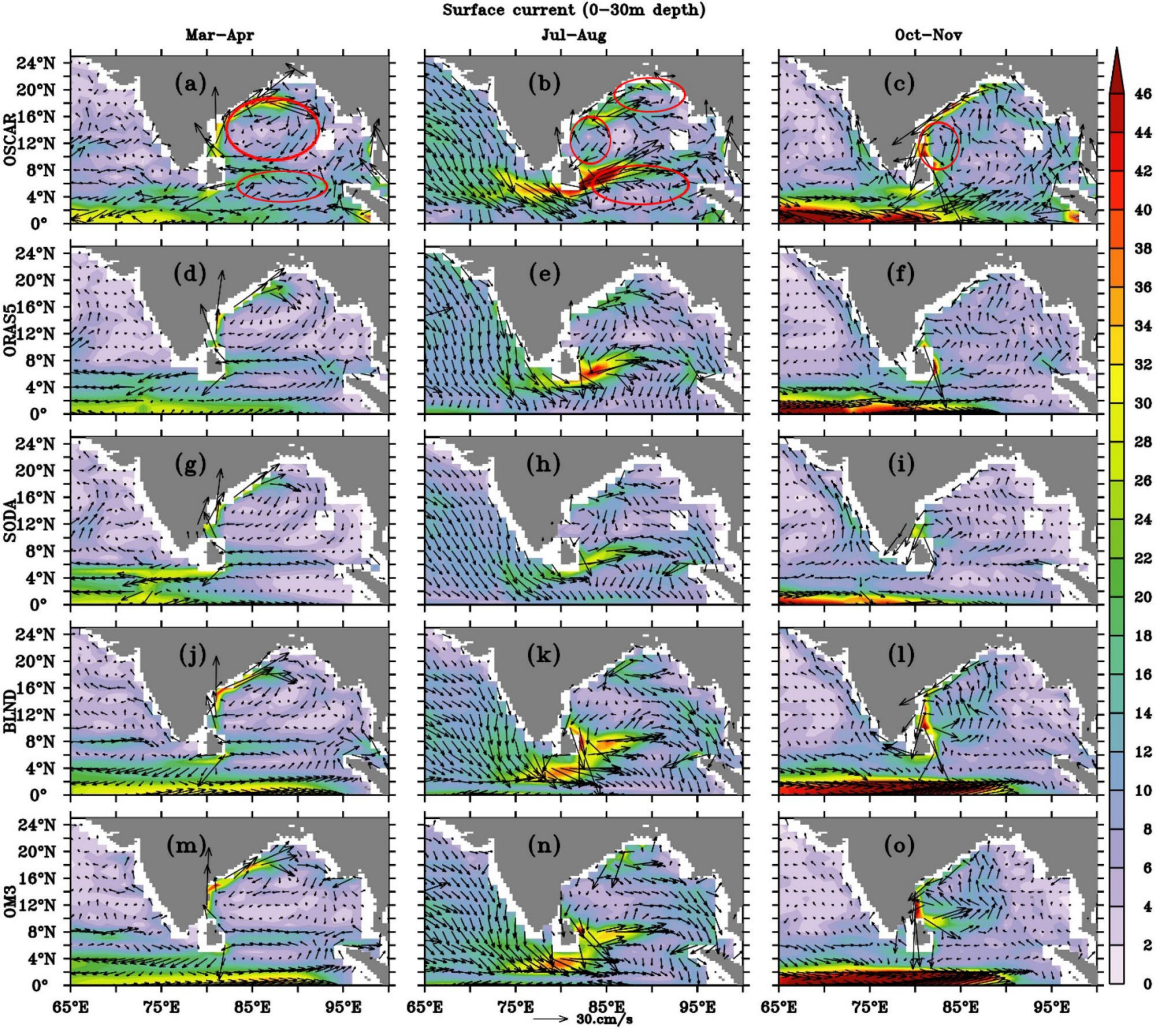
The mean surface current speed for the upper 30 m from 1992 to 2005 for (**a**–**c**) OSCAR, (**d**–**f**) ORAS5, (**g**–**i**) SODA, (**j**–**l**) BLND and (**m**–**o**) OM3 are shown in shade. The mean for the months of March–April (**a**,**d**,**g**,**j**,**m**), July–August (**b**,**e**,**h**,**k**,**n**), and October–November (**c**,**f**,**i**,**l**,**o**) are shown as the representative season for spring, summer, and fall. The arrows show the current vectors.

Coastal currents around the Indian peninsula change their direction with season^2,13–15,102^. The EICC flows poleward during February-May and equatorward during October-December. WICC flows equatorward during the Summer monsoon and poleward during the Winter monsoon^11^. Since the major changes in the current directions occur during inter-monsoon and monsoon periods, we show OM3 and BLND current (0–30 m averaged) comparison with OSCAR analysis during March–April, July–August and October–November along the coast of India and BoB in Fig. 7. We also included ORAS5 and SODA to assess how well the best reanalysis products reproduce the boundary currents along the coast of India and BoB. Figure 7 shows the surface currents (0–30 m average) in the BoB and eastern part of the Arabian Sea.

##### Spring currents

The BoB anti-cyclonic gyre (ACG), which is feebly present in late winter, becomes more defined in spring, with a poleward EICC arising because of the prevalent negative wind stress curl^103,104^. This observed anti-cyclonic gyre in Mar-Apr in OSCAR analysis is reproduced in all simulations. The western edge of the ACG is demarcated by a narrow, intense, and meandering northward flow, termed the Western Bay of Bengal Current (WBC) of the spring inter-monsoon period, also popularly known as EICC. Its magnitude is weaker in the reanalysis products and most accurately represented in BLND simulation (Fig. 7a,d,g,j,m) as compared to observations. It is worth noting that most of the observations do not represent the narrow band-like costal structure as seen in regional simulations. The poleward flowing EICC structure is most prominent in the regional simulation, but ORAS5 and SODA failed to capture the width and magnitude of EICC. This may be due to the unrealistic representation of bathymetry in these models, lack of observational data that went into the assimilation cycle, or forcing fields (Fig. 7a,d,g,j,m). The impact of bathymetry can be seen very prominently in EICC simulations. Accurate bathymetry representation in BLND simulations shows continuous EICC structure where as in OM3, it is not prominent along 80° E near 8–16° N. Rahaman et al.^98^ have also shown that ORAS5 and SODA reanalysis products are also not able to reproduce the observed EICC structures.

The observation shows a southern branch of ACG retroflecting at the southern tip of Sri Lanka and forming a cyclonic gyre (CG) marked as a red circle in Fig. 7a. The presence of this CG can be seen in ship drift observations as well (Figure not shown) and most prominently seen in OSCAR. The regional models, as well as ORAS5, reproduce this CG; however, SODA fails to reproduce this cyclonic gyre (Fig. 7g). The eastward-flowing CG component turns north when it encounters the coast of Sumatra and continues along the eastern boundary of the bay. This northward flow of the current, along with the inflow from Malacca strait, is captured in BLND simulations but weaker compared to OSCAR observation (Fig. 7j). This is almost absent in SODA and ORAS5 (Fig. 7d,g). It is worth mentioning that the Malacca Strait is open in the regional model, which is closed in the model used to generate the SODA and ORAS5 reanalysis products. This intruded water from the South China Sea through the Malacca Strait^105^, moves northward, following the Sumatra coast along the Eastern boundary of the bay up to 15° N. This current supplies water to the BoB ACG along with the northward flowing EICC in BLND, which is supported by OSCAR observation.

##### Summer currents

The summer monsoon current becomes prominent after the onset of the summer monsoon in early June^3,17,45^. Figure 7b,e,h,k,n shows the comparison of surface currents in July–August from ORAS5, SODA, BLND, and OM3 with the OSCAR observations. A strong eastward current (~ 30–36 cm/s) is seen on the southeast coast of Sri Lanka, which is notably underestimated (~ 20–24 cm/s) by both the reanalysis products ORAS5 and SODA (Fig. 7e,h). However, both the regional simulations accurately captured this current as compared to OSCAR observations (30–36 cm/s). Observation shows a cyclonic cell near the head of the Bay of Bengal during summer^106^. The presence of this cyclonic cell can be seen in OSCAR observations (see Fig. 7b) marked with a red circle. This cyclonic cell is very well reproduced in BLND simulation (Fig. 7k), and it is nearly absent in both the ORAS5 and SODA reanalysis products. Although its presence can be seen in OM3 (Fig. 7n) simulation, it is not very prominent. The reproducibility of this cell in BLND simulation shows the importance of accurate bathymetry representation in the ocean general circulation model. From the monthly analysis of currents, it was recognized that the signature of cyclonic circulation forming around 18° N in northern BoB during the summer monsoon months, as reported by Potemra et al.^107^, is not resolved in OM3 and only feebly present in the reanalysis products. However, it is comparable to that of OSCAR in BLND simulation (Fig. not shown). The WICC flows equatorward and, on reaching the southern end of Sri Lanka, turns northeastward to BoB (Fig. 7b). The WICC and northeastward current east of Sri Lanka attained their maximum intensity in July. The OSCAR observation shows the Arabian Sea water enters into the central BoB through the south and east of Sri Lanka^101^ with a maximum speed of 70 cm/s when the monsoon wind is the strongest and Ekman drift reaches its peak^17^. The maximum speed with which the Arabian Sea water enters the BoB is ~ 40 cm/s and ~ 30 cm/s in ORAS5 and SODA, respectively. However, in the regional simulations, it is ~ 32–36 cm/s in BLND and ~ 26–32 cm/s in OM3 simulations, which also underestimates the OSCAR observation (Fig. 7k,n). The enhancement of current speed in BLND simulations as compared to OM3 simulations can be considered entirely due to the more realistic bathymetry representation. After entering south BoB, the summer monsoon current flows towards the northeast and forms a CG. The presence of this CG over the southern BoB was reported by Varkey et al.^99^. The EICC during this time is disorganized^3^, despite the fact that the summer monsoon current is most developed^17^.

##### Fall currents

After the summer monsoon, in October–November, EICC reverses its direction and starts flowing equatorward^10,12,15,17,45,105^. This current is well simulated in regional model simulations as compared to ORAS5 and SODA reanalysis. The fall (October–November) current in the BoB is marked by the presence of a two-gyre system^107^. The BoB circulation shows a cyclonic gyre at the western part of the BoB, along the eastern coast of India (Fig. 7c) marked in a red circle. At the same time, the signature of a small anti-cyclonic gyre is observed in the northeast portion of the BoB. Both these gyres can be seen in all the simulations but are most prominently reproduced in the BLND simulation (Fig. 7l). The OSCAR observation shows the BoB water enters the Arabian Sea during this time through EICC. Observation shows that fall EICC carries BoB water as a narrow river^108^ along the east coast of India towards the equator. This narrow river-like path almost follows the shallow bathymetry along the coast. On reaching the south of Sri Lanka coast, it bends towards the west and flows northward as WICC, reaching above 20° N^31^. Only the BLND solution was able to simulate the narrow EICC as seen in OSCAR observation (Fig. 7l,c). This narrow EICC is very feeble in SODA and ORAS5 reanalysis (Fig. 7f,i). Even though the starting point of equatorward EICC is observed from 20° N in both the OSCAR analysis and BLND simulation, in the OM3 simulation, it initiates at 17° N (Fig. 7c,f,i,l). The observed WICC structure^14^ during this season is well captured by the reanalysis products and the BLND solutions (Fig. 7c,f,i,l). However, in OM3, it is completely absent (Fig. 7o). This result shows that bathymetry plays a major role in the realistic simulation of WICC. A westward flow at the southwestern tip of India, later moving towards the western coast of India because of the presence of Lakshadweep high, is simulated in regional models. In support of the model simulations, Chatterjee et al.^84^ reported in the North IO Atlas that the low salinity water spreads more westward off southwest India than poleward along the coast due to the existence of Lakshadweep high.

The strong northward/northwestward currents in the Andaman Sea and along the Myanmar coast shown in OSCAR (Fig. 7c) are greatly underestimated in both the model experiments (Fig. 7l,o). This underestimation is mainly due to the underestimation (over) of temperature(salinity) in upper 0–30 m over this region. The temperature in both the BLND and OM3 simulations is 1–2 °C cooler than the CORA observations over the Andaman Sea and along the Myanmar coast (Figure not shown). This could lead to a much weaker current over this region compared to OSCAR observation.

#### Upper ocean currents

##### Equatorial current from model simulations and observations

In this section, we show the upper ocean (40–200 m) currents over the EIO. We compared the seasonal cycle of zonal currents from regional model simulations and reanalysis products with ADCP observation. The seasonal cycle was computed from 2000 to 2005. During the monsoon transition period, i.e., spring (April–May) and fall (October–November), a strong eastward current named Wyrtki Jets (WJ) appears in the central EIO. These jets transport warm waters eastward from the western EIO to the eastern EIO in the upper ~ 100 m depth. This WJ plays an important role in the large-scale heat and freshwater transport in the tropical IO^2,12,109,110^. There are several observational^111–113^ and modelling^114–117^ studies focused on understanding its dynamics on seasonal and intra-seasonal time scales. Recently, Deshpande et al.^118^ have shown its inter-annual variability and its relation to Indian monsoon rainfall.

Observations show the presence of a subsurface Equatorial Undercurrent (EUC) just below the WJ over the central EIO, which is reported in various studies^2,103,119–122^. In the Pacific and Atlantic Oceans, the EUC is a quasi-permanent feature because of the prevailing easterly trade winds^123–126^. In the EIO, however, it is transient and depends on winds and pressure gradient variations associated with the distinct seasonal cycle due to the Asian monsoon. It is most pronounced in the Northern Hemisphere winter^122,127^, with its presence and absence mainly determined by the weaker and stronger easterlies in late winter and early spring^112,115^. The EUC is also present during the southwest monsoon but is much weaker compared to spring and fall^112^. It is associated with equatorial waves^127^ driven by the strong seasonally varying surface winds^2^. The core of this eastward undercurrent is located in the thermocline region^119^ above 300 m, beneath which a weak westward counter-flow exists and can last for at least a month during winter and spring. Observations show that the magnitude of the eastward undercurrent can reach 1 m/s during March-June and is comparable to the Pacific Ocean undercurrent magnitude, which exceed 1 m/s^123,125,126,128^. Unlike the IO EUC, which occurs during the inter-monsoon period, the EUC is a quasi‐permanent feature in the equatorial Pacific. Within the depth range of the thermocline, the EUC flows eastward from north of the New Guinea island to the west coast of the American continent^123,125,126^.

**Figure 8:**
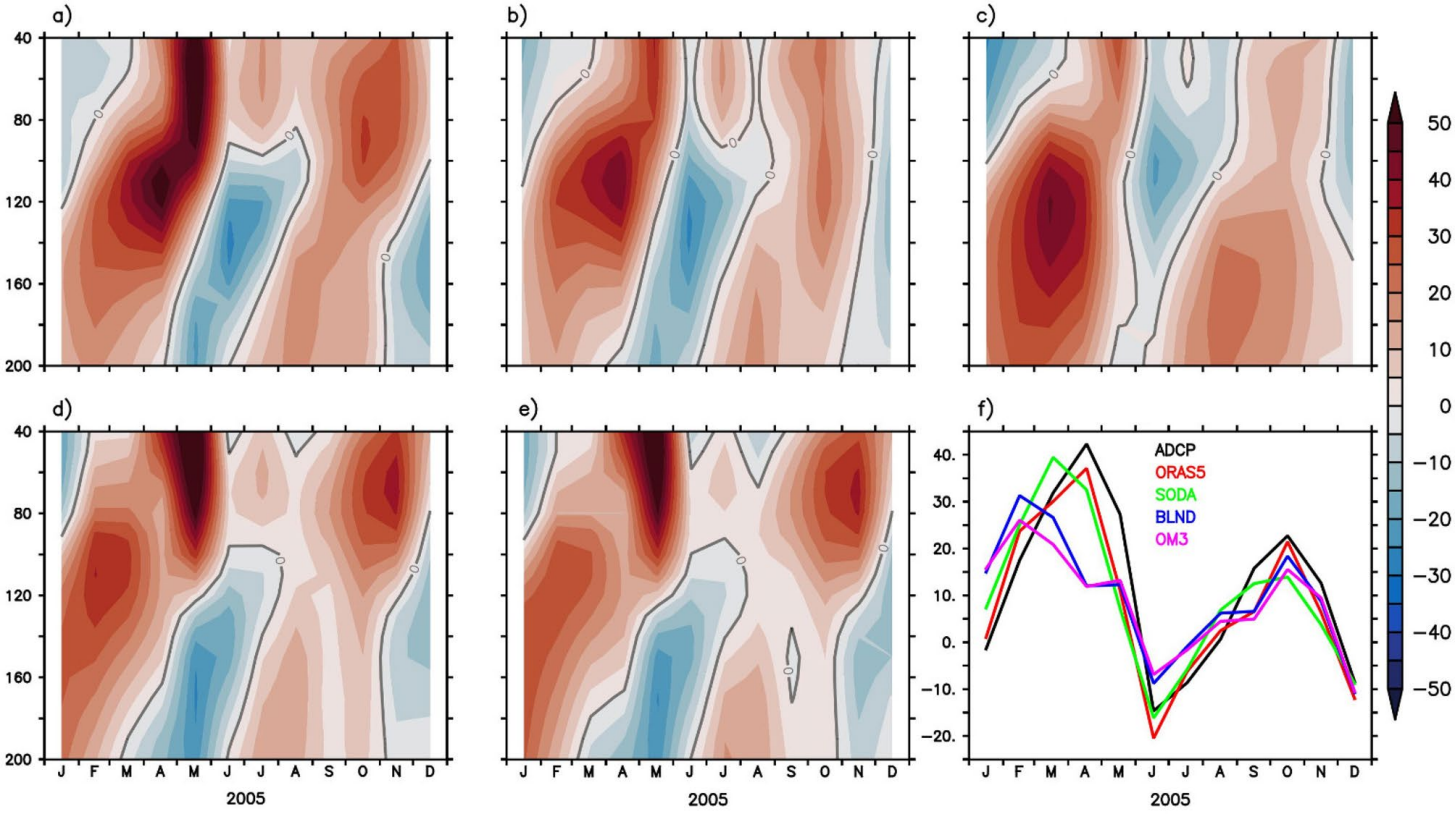
Upper Ocean (40–200 m) depth-vs-time mean (2000–2005) zonal current at 90° E, equator from (**a**) ADCP (**b**) ORAS5 (**c**) SODA (**d**) BLND and (**e**) OM3. (**f**) Shows the seasonal variation of zonal current averaged over 80–150 m depth from ADCP and all simulations at 90° E, Equator.

Previous studies show that forced model simulations are able to capture the undercurrent reasonably well^26,122,129^. A comprehensive evaluation of how WJ and EUC are represented in global models is still lacking. In this study, we show whether bathymetry plays any role in EUC simulations or not. We used ADCP observation to analyse the seasonal variation of current at 90° E, 0° N. The 5-year mean (2000–2005) of the ADCP data is compared with the regional simulations (OM3 and BLND) and reanalysis products (SODA and ORAS5). Figure 8a–e shows the upper ocean (40–200 m) zonal current at 90° E from ADCP observation, ORAS5, SODA reanalysis, and OM3, BLND regional model simulations. The ADCP observation shows a strong (sub-surface) jet named EUC forms with a core just below 100 m (within 80–150 m depth) at the beginning of March, which intensifies in April with a magnitude around 45 cm/s and weakens by the end of April (Fig. 8a,f). Just after the decay of this jet, WJ forms in the upper 100 m depth and peaks in May^3,109,112^, whose lower part is visible in Fig. 8a. The strong surface current along the equator is not seen during the peak monsoon period, July–August, even though the monsoon winds are strongest in July. The EUC again strengthens during October with much weaker strength as compared to spring time and decays slowly^3^ afterward. All the models are able to capture this observed seasonal variation, but it is underestimated. Neither of the reanalysis products could capture the WJ strength, and it is very much underestimated, with only 20–30 cm/s compared to the observed peak value of 60 cm/s. However, both the regional simulations are able to capture the strength of WJ (see Fig. 11). The seasonal cycle of EUC from ADCP and all the simulations is shown in Fig. 8f. The observed EUC peaks during March–April, but in regional simulations it is in February–March (Fig. 8f). Although the magnitude of the EUC is weaker in the regional simulation compared to ADCP and reanalysis, the impact of realistic bathymetry is evident. The EUC magnitude is improved in BLND simulations compared to OM3. The peak EUC value in OM3 simulation is ~ 25 cm/s, but in BLND simulation, it is ~ 30 cm/s. The reason for the early peak of EUC in the regional simulation is due to the inability of models to represent Kelvin waves realistically (See Rahaman et al.^26^ for more details).

##### Wyrtki Jet spatial structure

Previous studies showed that WJ extends up to a depth of 100 m^130^, and exerts zonal influence on the thermocline and sea surface height^131^, potentially impacting climate^47^. These jets play a crucial role in the zonal transport of water and associated properties^93,112,132,133^. However, the impact of bathymetry on WJ simulation remains unexplored. Also, the evolution of the spatial structure of WJ has not been reported in detail. Here in this section, we investigate the influence of bathymetry on WJ simulation using a nested regional ocean model.

**Figure 9:**
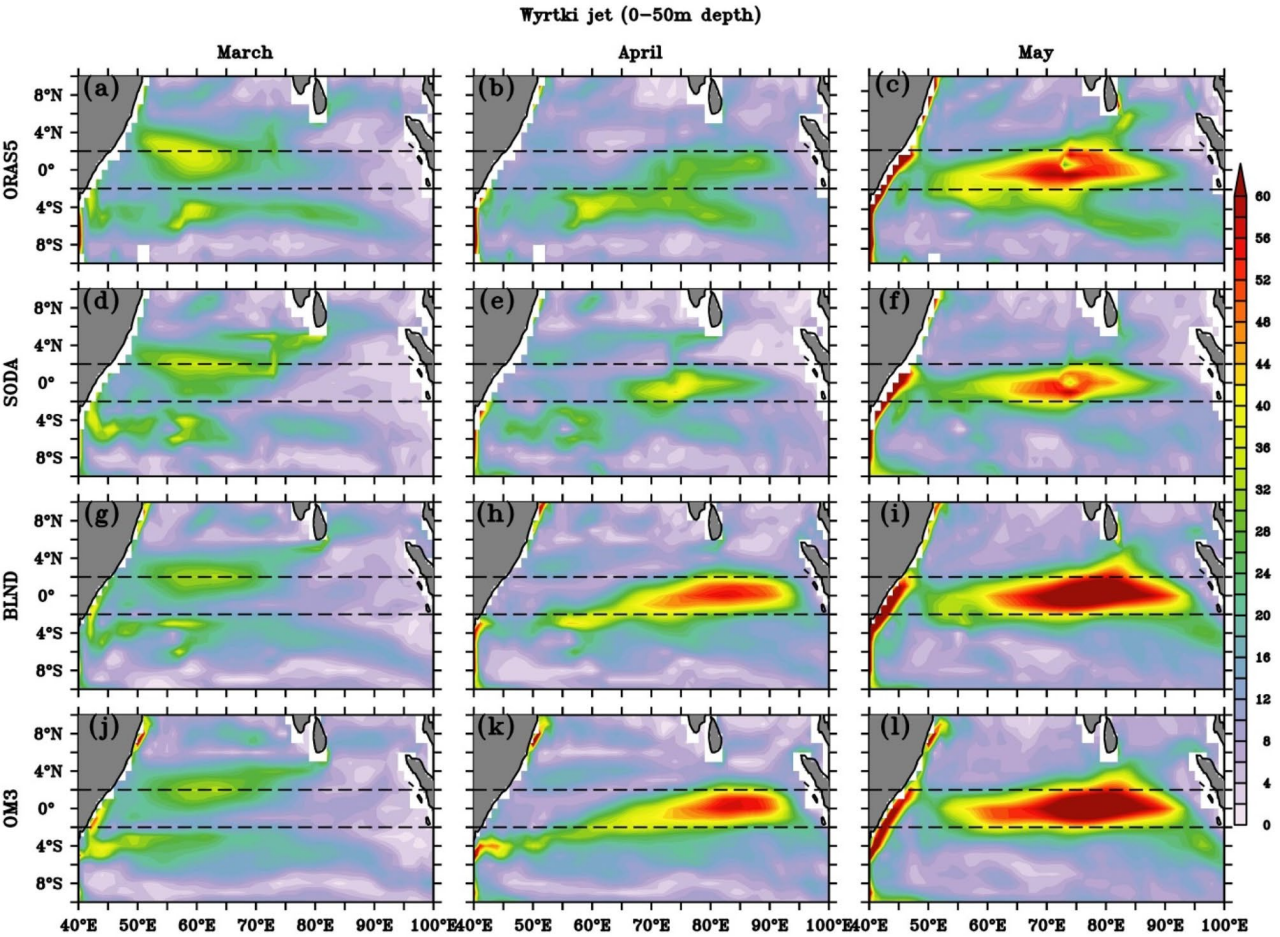
Upper ocean mean (1992–2005) current speed (cm/s) averaged over upper 50 m for (**a**–**c**) ORAS5, (**d**–**f**) SODA, (**g**–**i**) BLND, (**j**–**l**) OM3. The spring Wyrtki Jet for the months of March (**a**,**d**,**g**,**j**), April (**b**,**e**,**h**,**k**), and May (**c**,**f**,**i**,**l**) are shown. The black dashed line shows latitudes 2° N and 2° S.

In order to see the spatial structure of WJ, we show the current speed in the upper 50 m of the EIO (10° S to 10° N) from March to May, which corresponds to the spring WJ in Fig. 9. Most of the previous studies based on observation show that the core of spring WJ peaks in May and is located between 2° S and 2° N^117,131,132^. In this study, we also found that its longitudinal extend lies between 60–80° E, with maximum magnitude at 70–80° E, and is perfectly zonal since currents at this location are eastward (Fig. S1). Both the reanalysis products (ORAS5 and SODA) and regional simulations were able to capture this pattern, with the magnitude of the reanalysis products much underestimated as compared to the observation. However, both the regional simulation shows a stronger current (> 60 cm/s) in comparison with the observed ship drift currents, which is ~ 60 cm/s (see Fig. 1 of Nagura and McPhaden,^117^ and McPhaden et al.^132^). The BLND and OM3 simulation showed higher values ~ 70–75 cm/s in May, whereas ORAS5 and SODA showed ~ 52–54 cm/s. We also compared the speed with ship drift current (0–15 m) with all the simulations, and it shows the observed speed in May over the central EIO even reached ~ (70–75) cm/s, which is also captured by both BLND and OM3 simulations (Fig. S3). The comparison with ADCP observation averaged over 40–100 m depth at 90° E shows a maximum observed speed of 60 cm/s in May, whereas it is 30 cm/s and 20 cm/s in ORAS5 and SODA, respectively (Fig. 11). However, the regional model simulations show very close to the ADCP observation. In BLND simulation, it’s 56 cm/s, and in OM3, it’s 52 cm/s. A similar comparison at 80° E for 2005 shows ADCP 73 cm/s and in ORAS5, SODA, BLND, and OM3; the values are 65 cm/s,40 cm/s, 74 cm/s, and 77 cm/s respectively. It can be seen that BLND simulation is the closest to the ADCP observation.

The enhancement of current speed in BLND simulations compared to OM3 simulations shows the impact of realistic representation of bathymetry in the BLND experiment compared to the OM3 experiment. Previous studies showed that the spring WJ is mostly zonal near the equator^132–134^. This study also shows similar results since the current speed is more or less similar to that of zonal current (Fig. 9 and Fig. S1). The impact of bathymetry is not highly pronounced in the spatial plots in the regional simulations since, in both this model, 0–50 m depth bathymetry is not fully represented. However, there is a slight difference with the reanalysis products near the Maldives at 75° E. (Fig. 9 c,f,i,l and Fig. S1c,f,i,l) in which bathymetry representation is better than the regional simulation. In line with previous studies that have reported the peak of the WJ occurring in April-May^109,132^, this study also shows similar features. However, the magnitude of WJ shows drastic enhancement from ~ (20–30) cm/s in April to ~ (40–60) cm/s in May (Fig. 11). This may be attributed to the rapid development and completion of the WJ within a week once the winds have started to blow consistently^109^. None of these simulations show the presence of wake due to Maldives. This may be due to the fact that the minimum water depth set in these models is not at the surface, which reflects the presence of land. Hence, the signature of the bathymetry effect is not reflected in 0–50 m average currents. The impact of bathymetry is very prominent below 50 m and can be seen in EUC variability in the next section, as the creation of wake due to the presence of Maldives.

**Figure 10:**
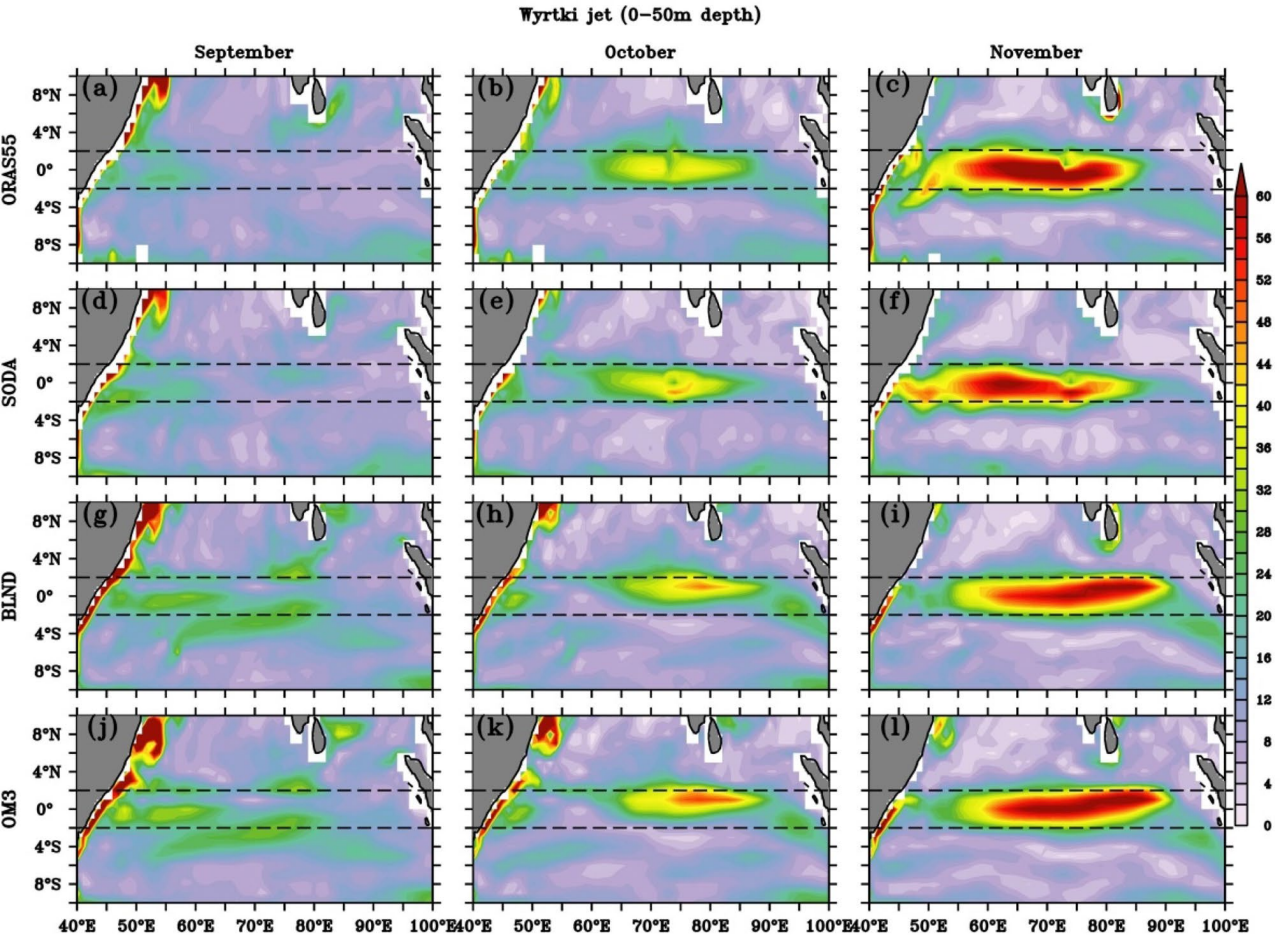
Spatial structure of Wyrtki Jet speed (cm/s) averaged over upper 50 m in (**a**–**c**) ORAS5, (**d**–**f**) SODA, (**g**–**i**) BLND and (**j**–**l**) OM3. The month of September (**a**,**d**,**g**,**j**), October (**b**,**e**,**h**,**k**), and November (**c**,**f**,**i**,**l**) shows the development of fall Wyrtki Jet. The black dashed line shows latitudes 2° N and 2° S.

**Figure 11:**
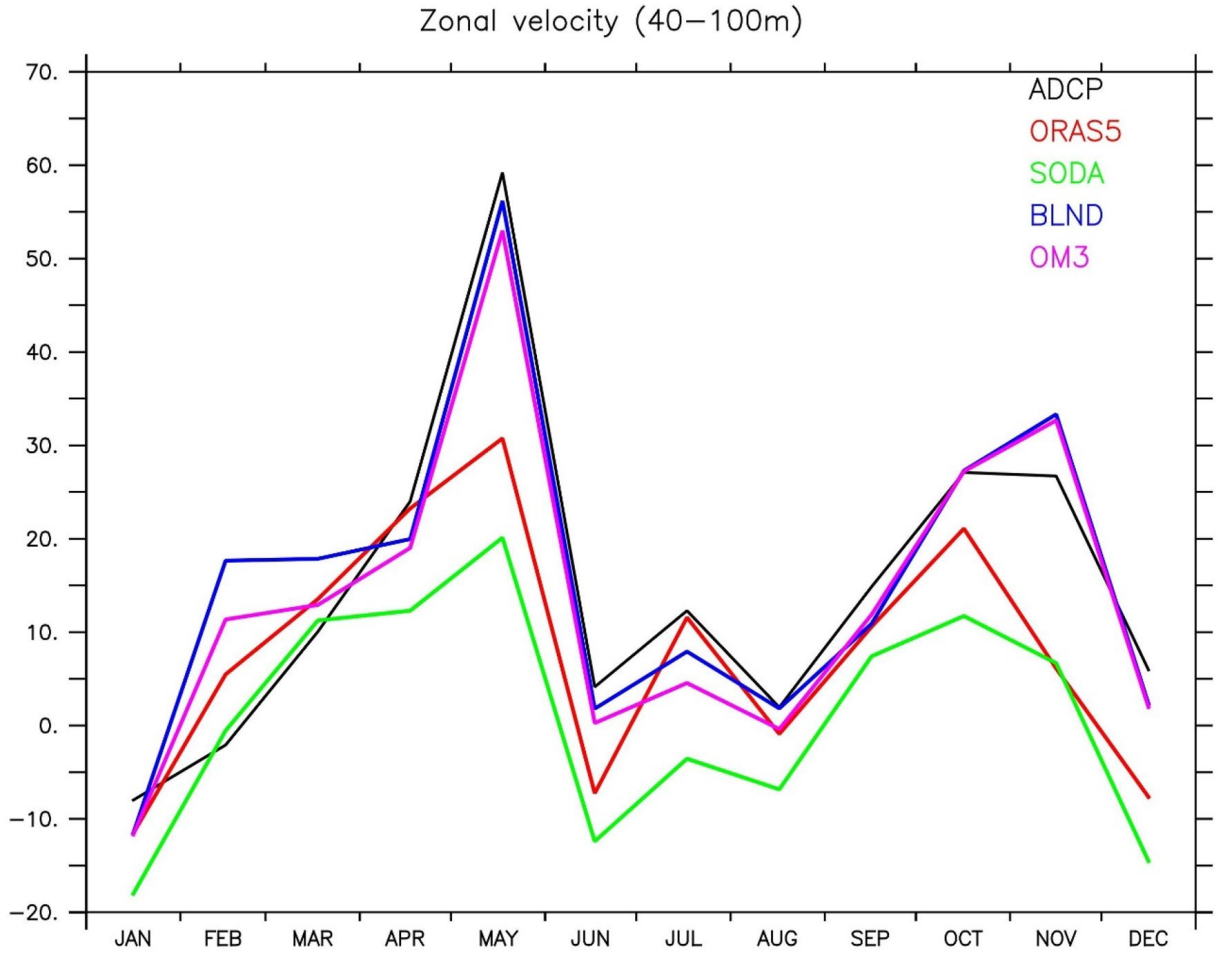
The seasonal cycle (2000–2005 averaged) of upper ocean zonal currents (cm/s) averaged from 40 to 100 m at 90° E.

Figure (9a–l) also shows a gradual north-eastward progression of eastward current from the southern to the northern hemisphere between March and May, with the dominance of eastward flow during the mature phase of WJ in the EIO (Fig. S1). Most of the previous studies showed the presence of fall WJ in the central and eastern EIO^132^. However, the spatial extent of fall WJ and its time evolution have not been reported. We show the evolution of fall WJ in Fig. 10, from all simulations. The spatial structure of fall WJ is similar to spring, and the zonal extent of the eastward current is more symmetric along the equator than in spring (Figs. 9, 10). The fall WJ is strongest in November, with speed > 60 cm/s, and extended almost the entire basin in all simulations, unlike spring WJ, which shows greater strength over the eastern EIO (Figs. 9, 10). Another striking difference in the spatial structure is that the fall WJ is more confined within ± 2 degrees of the equator and is symmetric. However, the core of spring WJ extended beyond ± 2 degrees of the equator and also more localized zonally. Like spring WJ, fall WJ is mostly zonal in nature, which can be seen with a similar magnitude of zonal current speed (Figs. 11, S2).

##### Equatorial under current spatial structure

**Figure 12:**
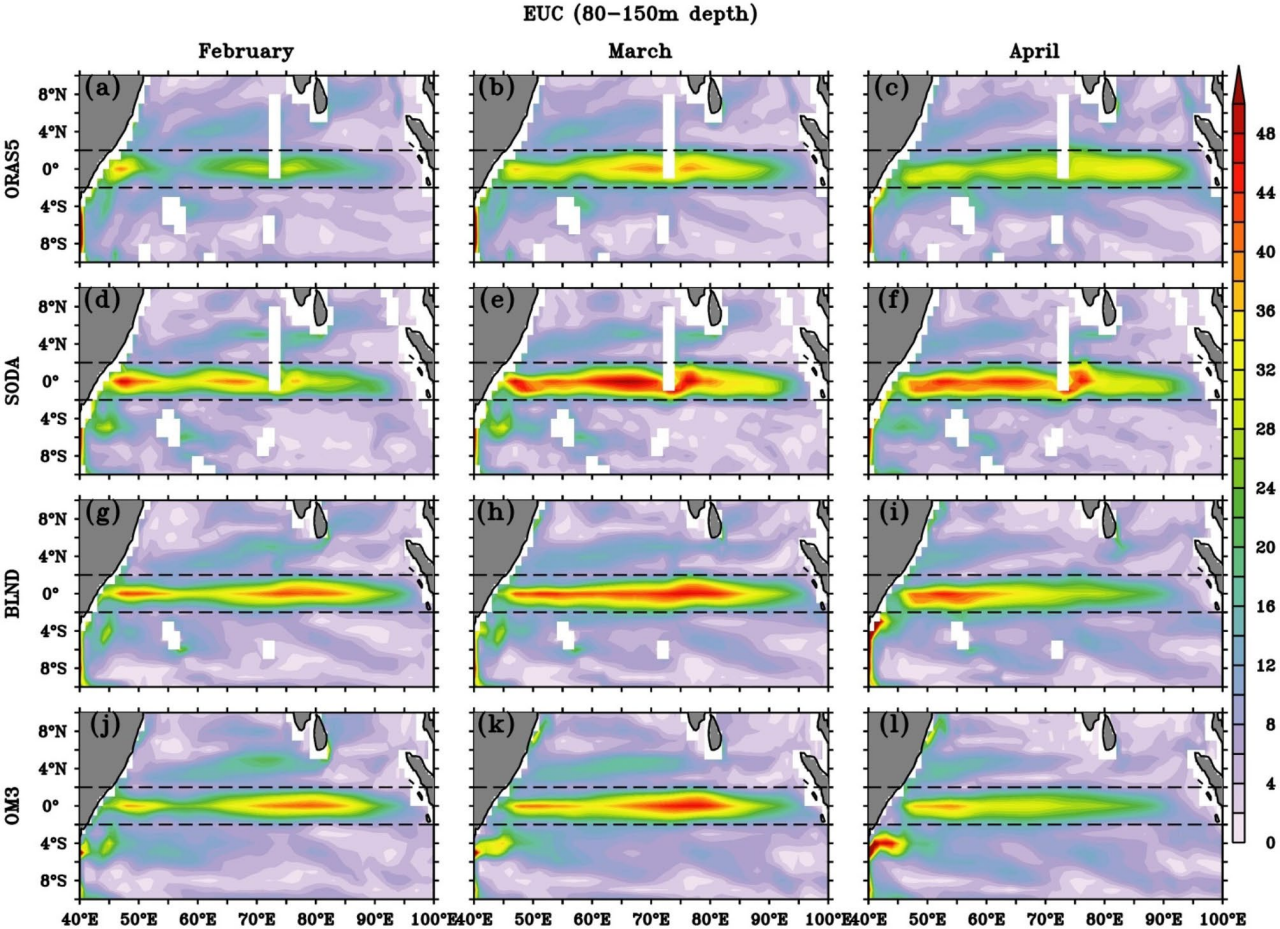
Spatial structure of Equatorial Under Current (cm/s) for the months of February (**a**,**d**,**g**,**j**), March (**b**,**e**,**h**,**k**) and April (**c**,**f**,**i**,**l**) for (**a**–**c**) ORAS5, (**d**–**f**) SODA, (**g**–**i**) BLND and (**j**–**l**) OM3 averaged over 80–150 m depth. The black dashed line shows latitudes 2° N and 2° S.

The EUC plays a major role in the zonal exchange of water, temperature, and salinity in the EIO^122,127^ and in sustaining upwelling at the eastern IO^135^. The spatial structure of EUC (80–150 m average) over the EIO from Feb to April^122^ from regional simulations and reanalysis products are shown in Fig. 12. It can be seen that the spatial extent of the EUC is spread almost throughout the EIO with meridional extent of ± 2 degrees. The meridional extent corroborates the findings reported by earlier observational studies, which show the presence of EUC at 55° E along the equator^132,136^. However, there are only observational studies that demonstrate the presence of EUC in the western EIO^119^. The zonal extent of EUC is more concentrated over the equatorial region and symmetric almost throughout the EIO, as compared to WJ (Fig. 9 middle panels and Fig. 12 right panels), which shows a narrower extent in the western EIO and wider extent in the central EIO.

**Figure 13:**
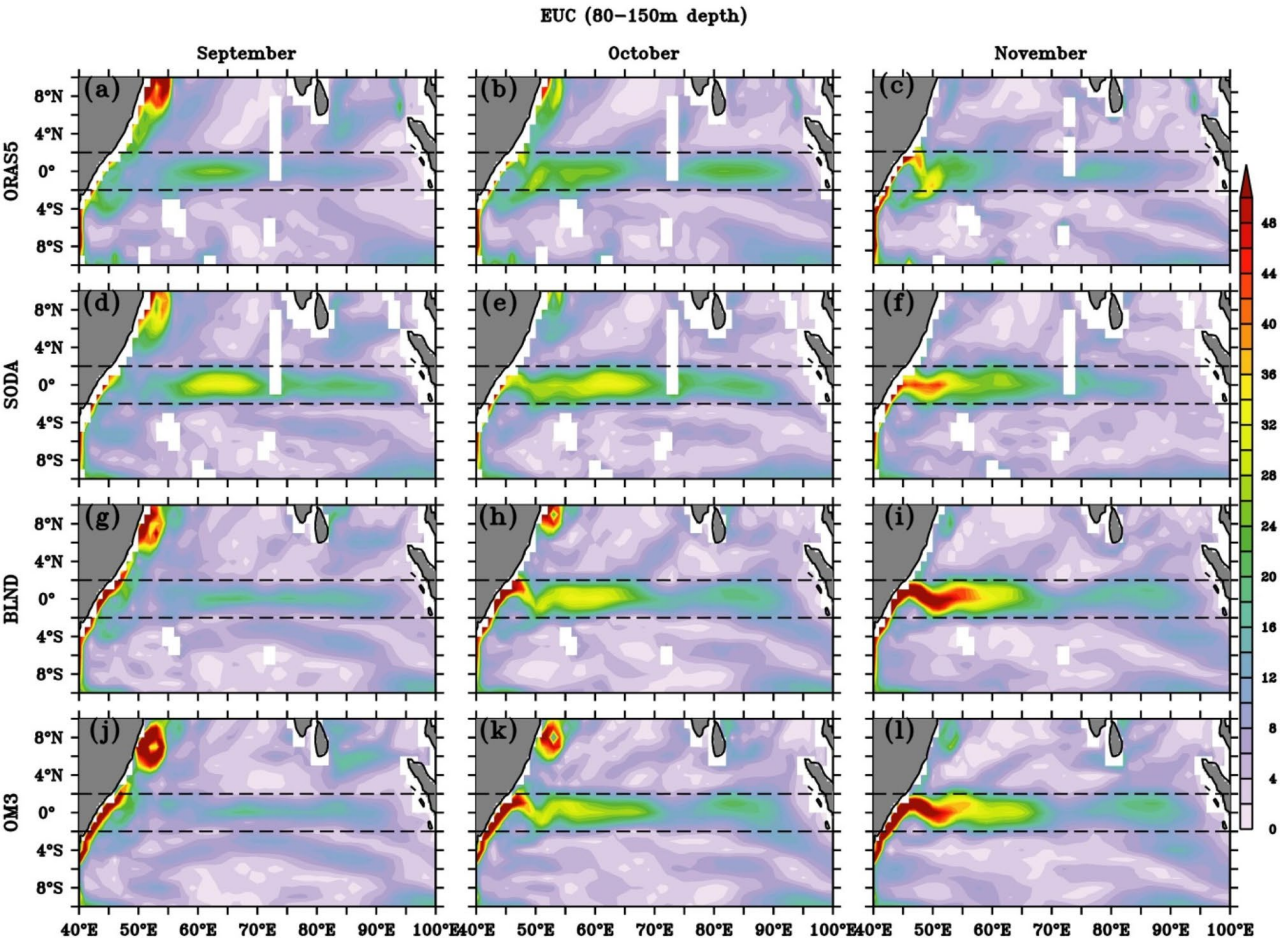
Spatial structure of Equatorial Under Current (cm/s) for (**a**–**c**) ORAS5, (**d**–**f**) SODA, (**g**–**i**) BLND and (**j**–**l**) OM3. The fall EUC for the months of September (**a**,**d**,**g**,**j**), October (**b**,**e**,**h**,**k**), and November (**c**,**f**,**i**,**l**) is averaged from 80 to 150 m depth. The black dashed line shows latitudes 2° N and 2° S.

As shown in Fig. 8, the EUC is most prominently seen at depths 80–150 m. Hence, we show the time evolution of 80–150 m average current speed in Fig. 12 from all the simulations during Feb-April. In all simulations, the Equatorial Undercurrent (EUC) magnitude peaks in March, with the maximum magnitude concentrated along 60–85° E. The EUC magnitude is weakest in ORAS5 and strongest in SODA. The presence of Maldives alters the currents in both the reanalysis products. However, in the regional models, the absence of Maldives does not result in significant deviation. It is worth mentioning that the average depth near Maldives is ~ 100 m in SODA and ORAS5 but is deeper in BLND (~ 200 m). In OM3, it is very deep (> 2000 m), and hence, Maldives is not represented in this solution (Fig. 12g–l). It can be seen in the BLND model simulations and reanalysis products (ORAS5 and SODA), that the EUC core is extended more towards the western EIO, whereas it is confined to central EIO in OM3 solution, in which complete Maldives is absent (Fig. 12h,k). This suggests the presence of the Maldives Islands is responsible for the westward extent of EUC. This is also reflected in the zonal current (Fig. S5 h,k). The presence of Maldives creates wakes, which is manifested as a wave, and its signature is very prominently seen in the SODA meridional current east of Maldives Islands (Fig. S6a,d,g,j). The presence of similar wakes has also been reported by Nagura and Masumoto^137^ for the WJ. We show here that EUC also creates wakes near the Maldives Islands. The intensification of the current on the leeward side, when obstructed by the Mascarene plateau, is noteworthy in all simulations except for OM3 (Fig. 12 and Fig. S5). The impact of bathymetry is most prominent near 45–60° E, where BLND simulation shows stronger EUC in April with a magnitude of ~ 50 cm/s, whereas in OM3, it is ~ 35 cm/s. EUC values in BLND simulation are similar to SODA and also corroborate the observed value of Swallow^136^. The EUC spatial structure during fall can be seen in Fig. 13. It can be seen that the core of the EUC in the fall season is situated slightly westward (50–70° E) as compared to the spring EUC, which is mostly present throughout the EIO (Figs. 12, 13, Fig. S5 and S7). OM3 simulation shows a narrower band than the slightly broader band in BLND simulation over the western EIO, also present in SODA.

**Figure 14:**
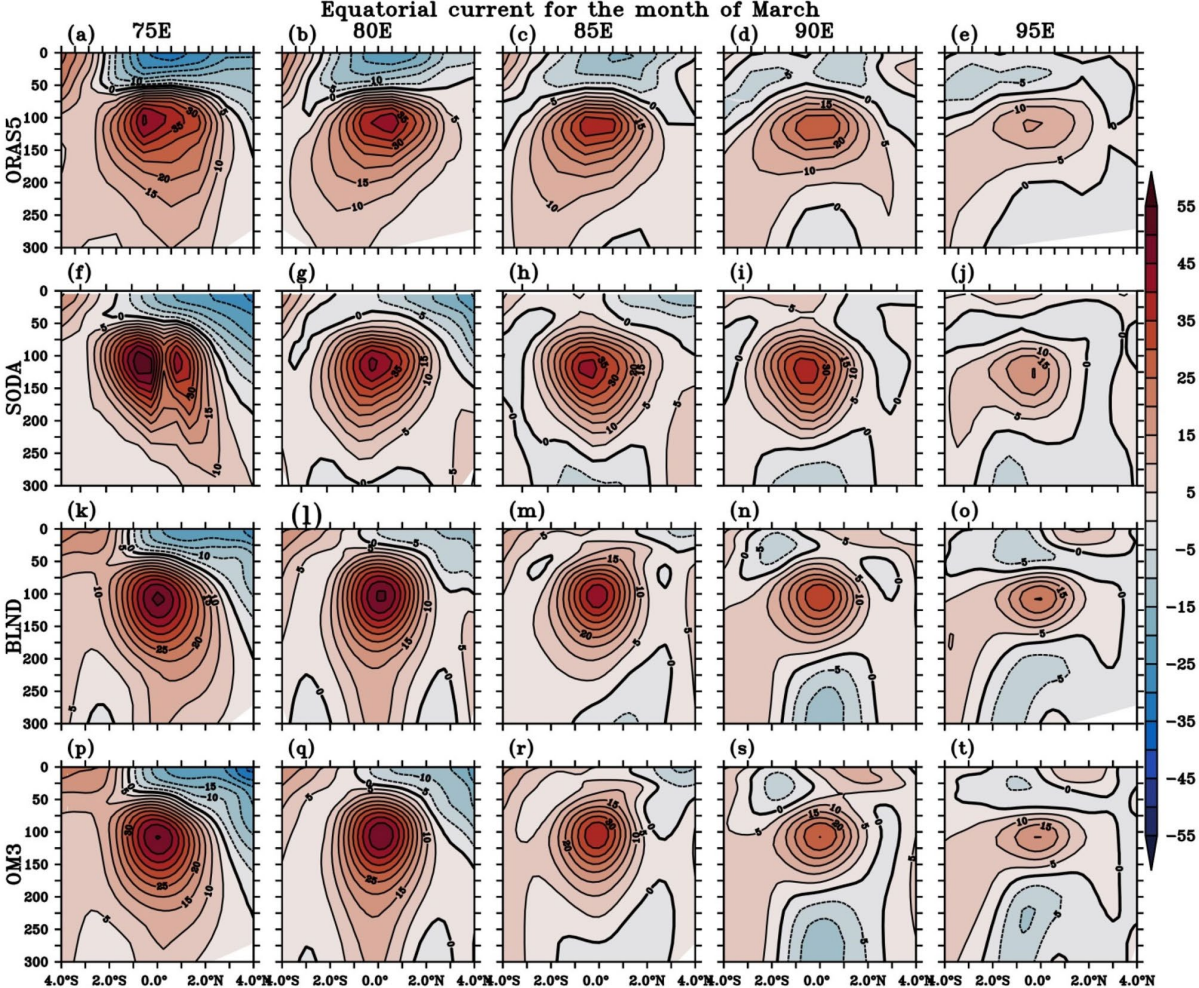
Depth-vs-latitude plot of zonal current speed at different longitudes (75° E–95° E) during the month of March is shown in shade. The overlaid black lines show the zonal current speed contour.

McPhaden et al.^132^, based on ADCP observation, showed that the zonal mean current is symmetric along the equator at 80° E. However, to our knowledge, no studies have reported whether EUC is symmetric throughout the equator or how its structure evolves with time for the entire equatorial region. In Fig. 12, we showed the depth-averaged spatial distribution of EUC, which is symmetric along the equator. To see whether EUC is symmetric with respect to the equator and the exact depth where EUC peaks, we show the depth versus latitude plots at different longitudes (75° E, 80° E, 85° E, 90° E and 95° E) centered along the equator in Fig. 14 for the month of March. Note that EUC magnitude is at its maximum in March for the entire EIO (Fig. 12b,e,h,k). It can be seen that EUC is strongest almost throughout the equator from 70 to 85° E and becomes weak at 90° E. The depth of the peak value lies around 100–120 m depth exactly at the equator. EUC structure is symmetric about the equator. The spatial and vertical extent of EUC is more in the western EIO, and slowly, it becomes narrower vertically and spatially towards the eastern basin extent. This structure is seen in all simulations with varying magnitudes of zonal current (Fig. 14). In April, similar features are seen (Fig. not shown). But, overall, the EUC became shallower and weaker spatially as compared to March (Fig. 14 and S8). Also, in both the regional simulations, the EUC structure is similar to SODA and ORAS5, but it’s slightly stronger at 75° E and 80° E. The impact of bathymetry in regional simulation is most prominent along 75° E and 80° E where the peak EUC value in BLND simulation is (50–55) cm/s, but it is (45–50) cm/s in OM3 simulation. EUC is much defined in March with a very prominent eastward zonal current at the thermocline. Chen et al.^129^ showed that the location of the EUC core varies along the longitude and with time. However, in this study, we show for a fixed location, the EUC strength is strongest in March, and in April, it is reduced. Also, by April, since WJ was also developed by this time, the signature of WJ was seen in the upper ocean (0–100 m) (Figure S8).

**Figure 15:**
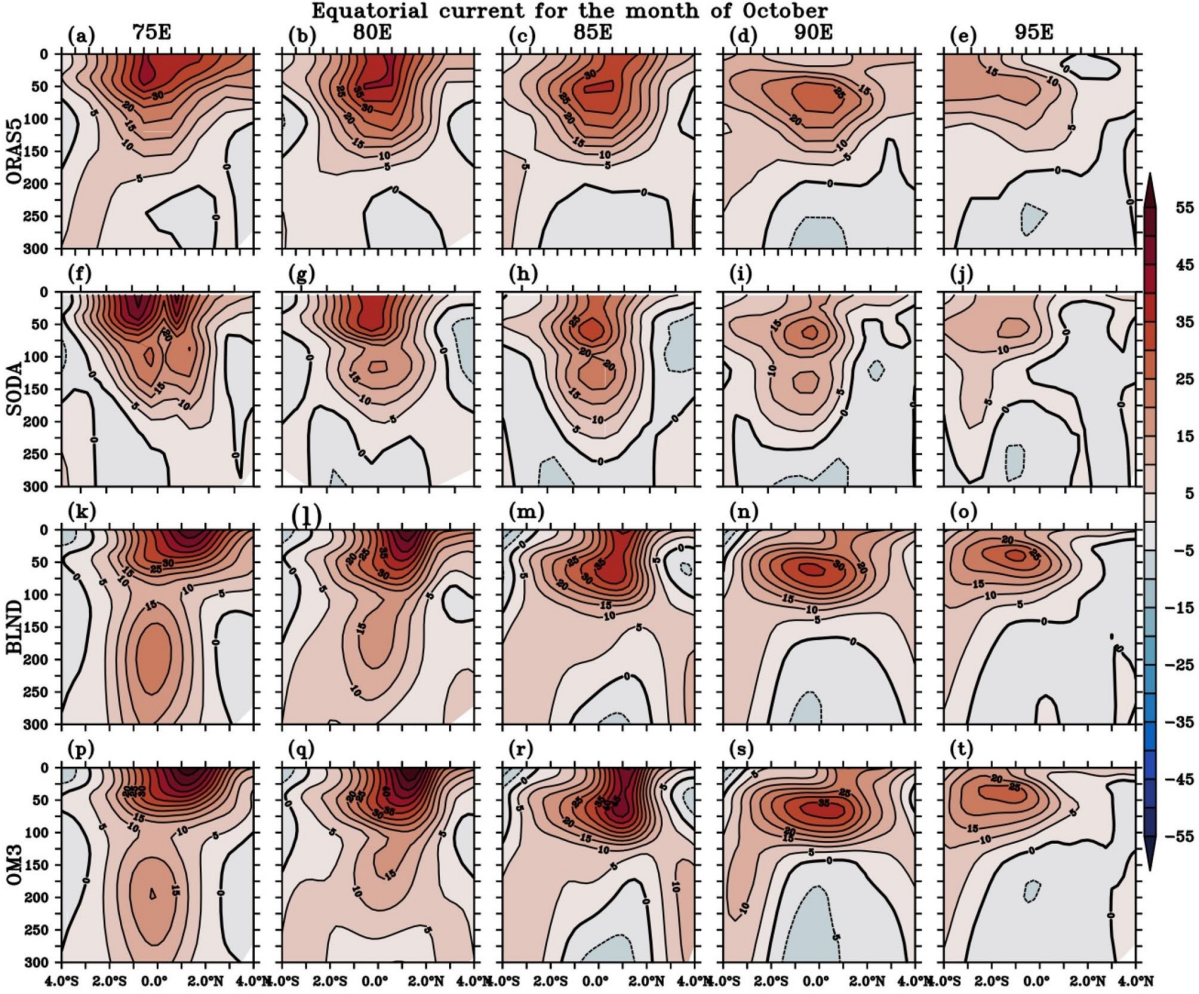
Same as Fig. 14 but, for the month of October.

In contrast to spring, when EUC structure is most noticeable in the western EIO (Fig. 14), during fall (Fig. 15), EUC is better defined in the eastern EIO 90° E and lies at a depth of between 50 and 100 m, unlike its spring counterpart, in which the core is located slightly deeper between 100 and 150 m (Fig. 14). The realistic bathymetry representation in BLND simulation enhances the EUC strength as compared to OM3 simulation (Fig. 15k–t). The fall EUC in ORAS5 (Fig. 15) shows a single core structure merged with WJ, and it is getting deeper and weakening towards the eastern EIO. All other simulations also show a similar structure except SODA, which shows a split double-cored structure along the equator at 75° E that penetrates to a maximum of 200 m depth. This splitting is due to the presence of Maldives Island in SODA (Fig. S6). The presence of Maldives also creates wakes on the leeward side, which is very prominently seen in the SODA meridional current (Fig. S6). The regional model simulations, on the other hand, show a double-cored structure with the EUC core centered roughly around 200 m deep (Fig. 15k–t) and the current reaching beyond 300 m depth at 75° E longitudes. The strength of EUC in regional simulation during fall is stronger than the ORAS5 and SODA reanalysis. This can also be seen in November (Fig. S9).

#### Intermediate and deep currents

##### Current at 1000 m depth

Even though the deep currents in the IO have been studied from the earlier days^138,139^ and further advancements in understanding have been made through the Second International Indian Ocean Expedition (IIOE-2), there is still a gap in understanding the intermediate and deep circulation of IO^133^. Several studies have been carried out to understand the deep circulation using model simulations^45,140,141^ and reanalysis products^142–144^. However, most of the studies were either related to meridional overturning or at specific locations. The spatial current patterns at intermediate and deeper levels have not been explored with the aid of observation or reanalysis data. In this section, we focus on the spatial pattern of currents at 1000 m depth and its dependence on the representation of bathymetry. To check the zonal pattern of currents at 1000 m depth, we compared the regional model simulation as well as reanalysis products with ANDRO data^145^. ANDRO is a Coriolis data product of velocity at Argo parking depths estimated from all Argo floats from 2000 to 2009. The zonal component of the ANDRO data shows a zonal striation pattern in the IO region (Fig. S10). The presence of this kind of structure was reported by Xia et al.^146^, with their dataset created from ARGO trajectories from 2004 to 2016. The model simulations at 1000 m depth also show a similar striation pattern in the zonal current in agreement with the Argo-derived velocity product, ANDRO (Fig. S10).

**Figure 16:**
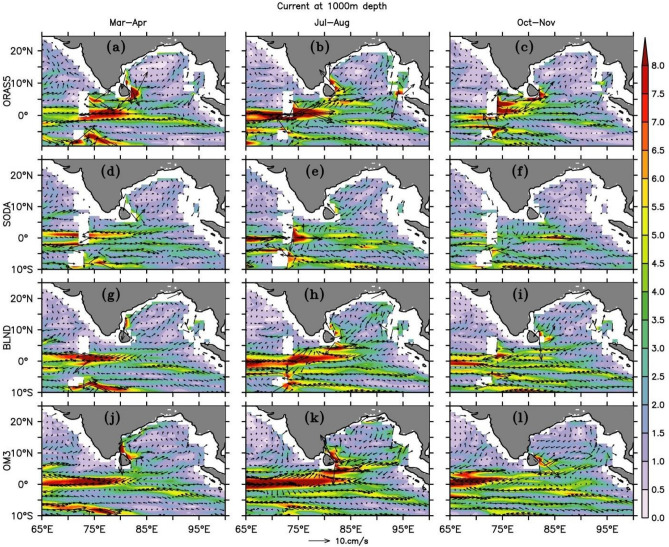
Currents at 1000 m depth for (**a**–**c**) ORAS5 (**d**–**f**) SODA (**g**–**i**) BLND and (**j**–**l**) OM3. The mean of March–April (**a**,**d**,**g**,**j**), July–August (**b**,**e**,**h**,**k**) and October–November (**c**,**f**,**i**,**l**) are shown as the representative seasons for spring, summer, and fall. Arrows show the current vector at 1000 m depth.

Figure 16 shows the intermediate current at a depth of 1000 m from all simulations for the spring, summer, and fall seasons. Since model levels do not precisely represent 1000 m depth, we took the 997 m depth level as 1000 m in SODA, and for ORAS5 (OM3/BLND), we took two levels averaged of 947.4 m and 1045.9 m (894.5 m and 1047 m). Significant differences can be seen in the bathymetry among all the models near ANI, Maldives, and Lakshadweep Islands. SODA represents the most realistic bathymetry, which shows the presence of Lakshadweep Islands; however, no other model shows these Islands. During inter-monsoon i.e., spring and fall, WJ like strong zonal jet is present at 1000 m depth but in the opposite direction, i.e., westward, unlike eastward in WJ. The presence of this zonal jet is seen in all simulations, with the strongest intensity found in ORAS5 at the central EIO (75–80° E). The magnitude of the jet ranges between 5 and 8 cm/s during spring among all the simulations, and is slightly weaker (~ 3–7 cm/s) during fall. The presence of Maldives reduces the magnitude of the jet in its west in all simulations. Nevertheless, in OM3, the jet is continuous due to the absence of Maldives (Fig. 16a,d,g,j). A clearer representation can be seen in supplementary Fig. S11.

The representation of the ANI at 1000 m depth, as illustrated in Fig. 16, exhibits variations between the model simulations and reanalysis products. These differences have implications for the portrayal of the Andaman Sea in the respective datasets. According to Hurlburt and Hogan^147^, the presence of smoothened bathymetry in model simulations raises concerns about potentially significant systematic errors in accurately representing the route and magnitude of deep circulation. This study further confirms this result. The impact of the topography in the model domain, particularly in the southeastern BoB, affects the direction and strength of the currents in the entire bay, with more significant consequences observed south of 12° N latitude. It is interesting that while there is an anti-cyclonic gyre at the surface during spring, the BoB shows a cyclonic gyre at 1000 m depth (Figs. 7, 16a,d,g,j). The presence of this cyclonic gyre is most prominent in BLND solutions and weaker in ORAS5 and SODA reanalysis products. Its presence is not prominent in OM3 simulation, probably due to the absence of ANI. The influence of bathymetry on deeper circulation, as emphasized by Hurlburt and Hogan^148^ regarding the strong impact of mean abyssal flow on pathways, becomes evident when examining the circulation patterns at a depth of 1000 m. During spring, the basin-wide gyre in the BoB and the formation of an anti-cyclonic gyre in the southern part of the BoB are influenced by bathymetric features. The intensity and structure of the cyclonic gyre in OM3 simulation differ significantly when compared with BLND simulation and reanalysis products (Fig. 16a,d,g,j). The EICC structure is prominently seen in all simulations along the coast of India. However, its direction is opposite, i.e., equatorward instead of poleward, as seen in the surface circulations (Fig. 16a,d,g,j). The narrow band of EICC, which is most prominently seen in the surface current along the east coast of India, is very prominently seen in the regional simulations with opposite directions of the surface. The magnitude of intermediate EICC is the strongest and most defined as compared to reanalysis and OM3 simulations. The presence of high-speed current near southwestern BoB along the coast of India in the BLND simulation is part of the cyclonic gyre G1 mentioned by Varkey et al.^99^ The magnitude is also comparable to the observed value ~ 17 cm/s reported in Varkeley et al.^99^. The Andaman Sea which is most prominently represented in BLND simulation shows a prominent ACG in all season (Fig. S12g,h.i) The presence of this permanent ACG has been reported by Varkey et al.^99^ at 1000 m depth from observation.

**Figure 17:**
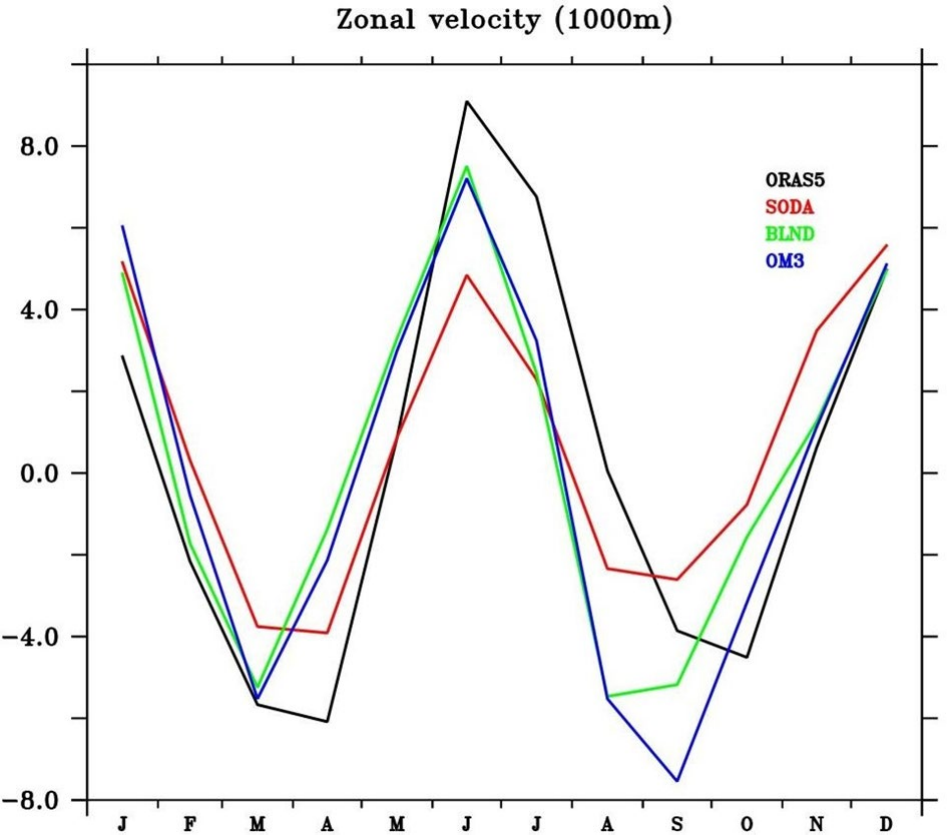
Seasonal cycle of zonal current at 1000 m depth averaged over the central equatorial Indian Ocean (75° E–85° E, 2° S–2° N), for ORAS5, SODA, BLND, and OM3 simulations.

During peak southwest monsoon season, the strong eastward current is present in all simulations at the east and south of Maldives (Fig. 16b,e,h,k, Fig. S11b,e,h,k), within 2 degrees centered at the equator. At the surface, the current north of 2° N is the monsoon current, and that south of 2° S is the South Equatorial Counter Current (SECC). The extent of SECC is very prominently observed in all simulations at 5–10° S at 1000 m depth. Recently, Chen et al.^149^ have named the subsurface SECC as the South Equatorial Undercurrent (SEUC). The SEUC exists across the basin from about 60° to 105° E, with a core centered around 8°–10 °S and vertically extending from 200 m to more than 2000 m. The seasonal cycle of zonal current at 1000 m depth averaged over central EIO (75–85 °E and 2° S–2° N) shows very prominent seasonality with eastward current in summer, which peaks in June and westward current during early spring (Mar–Apr) and early fall (Sep–Oct) (Fig. 17). This structure is just opposite to that of EUC (see Fig. 8f). The presence of Maldives retroflects the current slightly northward and then again eastward (Fig. 16b,e,h,k, Fig. S11b,e,h,k). This signature is prominent in all simulations except OM3. Due to the absence of the Maldivian islands in OM3, a continuous eastward intensified current is observed in the south of Maldives Island (Fig. 16k, Fig. S11k). Similar to the surface and subsurface equatorial current, the deep currents also show jet-like structure, especially to the east of the Laccadive ridge. Currents tend to get magnified at the leeward side of a bathymetric barrier; the phenomenon is evident in the intensification of eastward current at the Laccadive ridge during monsoon at the equator. Also, the strengthening of the westward current at 4° S following a bathymetric obstruction at the same time (Fig. 16 and S12b,e,h) is also observed in all simulations. Warren^150^, in his study using CTD data, detected a zonal jet at 90° E (north of 10° S) ridge from the overflowing water at 4300 m depth that actually fuels the western boundary current.

During fall, unlike surface circulation, which shows a much weaker current over the eastern BoB and ANI region at intermediate depth, the regional simulation shows a stronger current. Also, unlike spring, when the EICC structure is very prominent, it is completely absent during fall in all simulations. The differences in the bathymetry representation in the regional simulations are reflected in the BoB currents. The Andaman Sea anti-cyclonic gyre is absent in OM3 simulation (Fig. S12j–l), which is prominently seen in BLND simulation (Fig. S12g,h,i).

##### Current at 2000 m depth

In the study conducted by Toole and Warren^151^, from a transoceanic hydrographic section across subtropical southern IO, it was found that the net transport below 2000 m is negligible. In agreement with their results, a later study done by You^152^ on the implication of deep circulation of the IO and water mass pathways found that the results at the 2000 m reference level give more details than 4000 m. In reference to the previous studies^151–153^, we analyse the deep ocean circulation at 2000 m over the eastern EIO and BoB.

**Figure 18:**
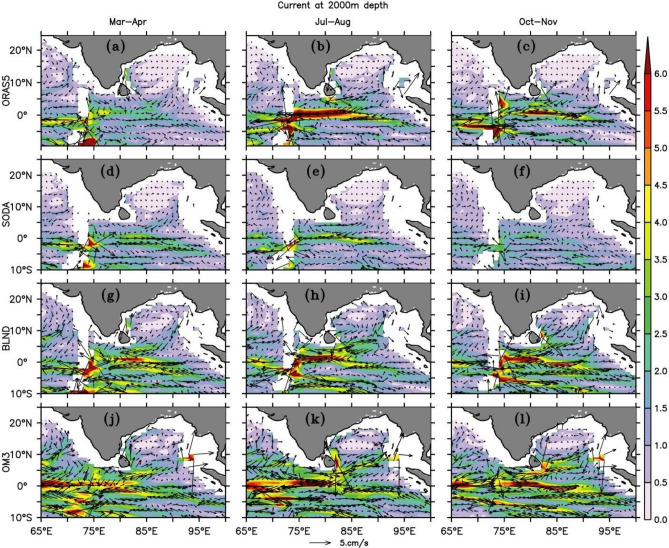
Same as Fig. 16 but for 2000 m depth.

The seasonal spatial variations of currents at 2000 m from all simulations are shown in Fig. 18. Although the reanalysis products show almost no motion in the BoB, regional model simulations show a noticeable circulation pattern in the BoB basin. The EIO shows a similar pattern of 1000 m current but with a reduced current speed. A Stronger westward current at the central EIO during summer (Fig. 18b,e,h,k; Fig. S13b,e,h,k) can be seen in all simulations. Regional simulations show more structure in the EIO in comparison with reanalysis products. Jain et al.^154^ observed an equatorial current with a climatological mean of ~ 3 cm/s during May–June at a depth of 2000 m, based on ADCP data moored at 83° E (see their Fig. 2b). Further investigation into the zonal currents at the same depth, in comparison with Jain et al.^146^ revealed that in the BLND regional model simulation, the deeper currents at 2000 m near the equator at 83° E have a magnitude ~ 4 cm/s which is slightly overestimated, and in SODA it is similar to the ADCP observed values. ORAS5 and OM3 underestimate the ADCP observation.

In the interior BoB, both the reanalysis products show very weak currents. In ORAS5, a feeble presence of EICC can be observed, which is absent in SODA. Similar to the WJ and EUC, regional simulations show a stronger current in the BoB. During spring, the equatorwards EICC is even present at a depth of 2000 m, which was also present at a depth of 1000 m (Fig. S14). At the deeper layer, the presence of equatorward current along the western coast of ANI can be seen in spring and fall with speed ~ 2–3 cm/s in BLND simulation, which is absent in all other simulations except in ORAS5 in which its feeble presence is noted. During summer, its direction reverses and flows in the northeast direction. The basin-wide differences in the current pattern can also be seen in BLND and OM3 simulations due to the differences in bathymetry in these two simulations.

## Summary and conclusion

Bathymetry influences ocean circulation in several ways; currents are modified near the coast, and even relatively small ridges on the sea floor can influence the direction of major ocean currents. In the deep ocean, ridges serve as solid barriers separating bottom waters in adjacent ocean basins. Hence, stand-alone ocean general circulation models and climate models are sensitive to the accuracy and resolution of the large-scale bathymetric representation while developing the models. Accurate representation of bathymetry also requires the effective implementation of mixing parameterization schemes in the model. In this study, we tried to show the impact of IO circulation by representing two different bathymetry in a regional IO model. While sensitivity studies of this kind are available for other oceans, there is a lack of similar studies specifically focused on the IO. In this study, we used two regional IO MOM4p1 model experiments that differed only in bathymetry to understand the effect of bathymetry in IO simulations. In one experiment, we used much-smoothed bathymetry, often used in the global ocean model named OM3, and in another experiment, we used more realistic bathymetry, particularly modified near the Indian coast named BLND. The numerical model with modified bathymetry (BLND) showed improvements in seasonal cycle and mean in upper ocean temperature, salinity, and ocean currents, especially near the coast, as compared to OM3 simulations. The upper ocean (0–200 m) temperature bias along the coast of India and the Myanmar coast is > 5 °C in OM3 simulations when compared with CORA observations. However, this bias is significantly reduced in the BLND simulation, which closely reproduces the spatial pattern observed in CORA data. The upper ocean (0–200 m) spatial mean salinity distribution in BLND simulation resembles the observed patterns. The low salinity coastal distribution is almost reproduced BLND. However, OM3 simulation was unable to capture the observed salinity distribution, particularly near the coast, and exhibited higher salinity when compared with CORA observation.

The realistic representation of bathymetry led to improved surface current simulations when compared with OSCAR analysis. EICC and WICC are more realistically reproduced in regional simulations. Both these currents are more defined in the BLND simulation than in OM3, which uses smooth bathymetry. The observed summer monsoon current pattern is most realistic in the BLND simulation as compared to all other simulations. To evaluate the regional models’ current simulations, we also compared them with reanalysis products, namely ORAS5 and SODA. Both the reanalysis products underestimated the EICC, WICC coastal current, and summer monsoon current as compared to the OSCAR analysis. EICC structure during fall in ORAS5 and SODA is nearly absent. The representation of bathymetry near the Indian coast, deficiency in model physics, or forcing field errors may have led to the near absence of the EICC core structure in SODA and ORAS5 reanalysis products. As explained in previous sections, the improvements in BLND simulation as compared to OM3 simulations are due to the more realistic representation of bathymetry since all other setups and forcing are the same for these two simulations. The main reason for this improvement can be attributed to the bottom-enhanced vertical mixing parameterization scheme in ocean general circulation models, which parameterizes diapycnal mixing over rough topography where stratification exists at depth. This study shows that despite high vertical resolution, diapycnal mixing parametrization does not effectively work on smooth bathymetry.

WJ at the surface layer and EUC at the thermocline in the EIO, which are two dominant currents, show semi-annual variations with peaks in spring and fall. The EIO is marked by the presence of Central Indian Ridge, Chagos-Laccadive Ridge, 85° E Ridge, and 90° E Ridge. These ridges are represented differently in both bathymetry used in the two regional simulations in this study. The OM3 shows much smoother bathymetry, in which Chagos-Laccadive Ridge is almost absent, and the other ridges are also not realistically represented. On the other hand, in the BLND simulations, most of the ridges are realistically represented. This is also true for the ORAS5 and SODA reanalysis in which bathymetry is realistically represented. In order to see how bathymetry influences the equatorial currents, we compared the regional model as well as reanalysis simulations with ADCP observation at 90° E. The strength of the EUC is most realistically reproduced in ORAS5 when compared with ADCP observations. The 5-year (2001–2005) mean EUC velocity observed by the ADCP during its peak in April is approximately 40 cm/s. In SODA and BLND, it is ~ 35 cm/s. In OM3 simulation with much smoother bathymetry shows weaker EUC (~ 25 cm/s). But in BLND simulation, it is ~ 30 cm/s. This improvement is only due to the realistic bathymetry representation in BLND simulation as compared to OM3 simulation. It is to be noted that in the regional model, the peak EUC appears two months early, in February instead of April, as compared to ADCP observation. Rahaman et al.^26^ have shown that this kind of early peak in the free-forced ocean model is due to the inability of these models to represent the observed Kelvin wave.

The space time evolution of the Upper Ocean current reveals that the WJ is zonal, which was also reported in earlier studies^132^. This study further shows that the extent of spring WJ is most prominent in May along 70–80° E, over the central EIO, and is affected by the presence of Maldives Island. Regional models show stronger WJ and the absence of Chagos-Laccadive Ridge and Maldives Island weakens the WJ. The BLND simulation almost reproduced the strength of WJ when compared with ADCP and ship drift observations. OM3 simulation slightly underestimates WJ, whereas in ORAS5 and SODA reanalysis, it is underestimated significantly. The fall WJ is most prominent in November, and its spatial extent is more confined towards the western EIO and symmetric along the equator with almost basin-wide presence (60–90° E). However, spring WJ is broader towards the eastern EIO and narrower in the central EIO. Also, spring WJ spatial extent is centered around 70–80° E.

The ADCP observation shows that EUC peaks at a depth of ~ 80–140 m. The time evolution of the spatial zonal current distribution of EUC averaged over this depth shows its prominent presence throughout the EIO during Feb- April. Although ADCP observation at 90° E shows its peak in April, the spatial distribution of zonal currents from the regional and reanalysis simulations show it peaks in the month of March along 65–80° E. EUC is symmetric along the equator, unlike spring WJ, which is narrower in the western and broader in the eastern EIO. The latitudinal variation of EUC at 75, 80, 85, 90, and 95° E shows that EUC is symmetric across the equator. Its structure is broader in the western EIO, and gradually, it became narrower and weaker towards the east during spring. In fall, EUC is most prominent in October, and its presence is noticeable over 85–95° E, with a much weaker magnitude than spring EUC. Although there was not much improvement in the magnitude of the Wyrtki Jet (WJ) simulation in the BLND simulation, it was observed that the spatial structure of both the WJ and EUC was affected by the presence of bathymetric obstructions in close proximity to topographic changes. These obstructions often triggered instabilities on the leeward side of the flow in the form of wake. The occurance of wake due to the presence of Maldives Island in the WJ has been reported in earlier studies^137^. In this study, we further show the presence of wake in the subsurface due to EUC, which is most prominent in SODA reanalysis.

The effect of bathymetry on circulation is most evident in the deeper currents. The absence of the ANI at the eastern part of the BoB significantly affects the current over the entire bay at 1000 m depth. We found the presence of a basin-wide cyclonic gyre at 1000 m depth during spring, which is just opposite to that of the surface circulation which shows the presence of a basin-wide anti-cyclonic circulation. The structure and intensity of the cyclonic gyre formed in spring differ when these islands are absent. The absence of ridges and plateaus also results in unrealistic continuous strong currents that would otherwise be topographically steered and modified zonally at obstructions in the EIO. We found the presence of a strong, narrow eastward zonal jet structure similar to the WJ at this intermediate depth (1000 m) in the central EIO. This is most prominent during summertime. The simulations show the presence of this narrow jet with peak values in June, with varying magnitude. Like WJ, the direction of this jet reverses during spring and fall, when it is westward. To the best of our knowledge, both the presence of basin-wide cyclonic gyres in the BoB and WJ-like eastward jets during summer in the EIO at 1000 m are the first results we are reporting. The circulation at 2000 m shows almost no motion in the interior BoB in both the reanalysis products. However, the regional simulations still show the cyclonic gyre, which was prominent at 1000 m depth. Along the western flake of ANI, the BLND simulation shows a noticeable counterflow, which is absent in all other simulations. The presence of equatorial jet seen at 1000 m is also present at 2000 m, but with reduced speed. The presence of EICC during springtime is even seen at 1000 m and 2000 m depth but in the reverse direction to that of surface EICC. Like surface, in the intermediate and deeper depth also EICC is most defined and strong in BLND simulations as compared to other simulations. Similar to the surface, at 1000 m depth also in both the reanalysis products, the EICC structure is weaker and broader as compared to BLND simulation. At a depth of 2000 m, the EICC structure during spring is completely absent. This study also found the first of its kind about the presence of a narrow boundary current along the western boundary of ANI at 2000 m.

This study demonstrated that realistic bathymetry representation is essential for the realistic simulations of surface and subsurface temperature, salinity and current variability. This study also found that two of the most widely used reanalysis products i.e., SODA and ORAS5, underestimate the WJ and boundary currents along the coast of India.

### Supplementary Information

Supplementary Figures.

## Data Availability

The datasets used and/or analyzed during the current study are available from the corresponding author on reasonable request.
