# Supplementary material for: Impact of bathymetry on Indian Ocean circulation in a nested regional ocean model
Source: Sci Rep. 2024 Apr 5;14:8008. doi: 10.1038/s41598-024-58464-2 (PMC10997594; doi:10.1038/s41598-024-58464-2)
Supplement: Supplementary file 1 — Supplementary Figures. [file 41598_2024_58464_MOESM1_ESM.docx]

**Impact of bathymetry on Indian Ocean Circulation in a nested regional ocean model**

Raheema Rahman^1,2^ and Hasibur Rahaman^1*^

^1^Indian National Centre for Ocean Information Services (INCOIS) Ministry of Earth Sciences(MoES), Government of India, Hyderabad, 500090, India

^2^KUFOS-INCOIS Joint Research Centre, Kerala University of Fisheries and Ocean Studies, Panangad, Ernakulam, Kerala, 682506, India

**Supplementary Figures**

**
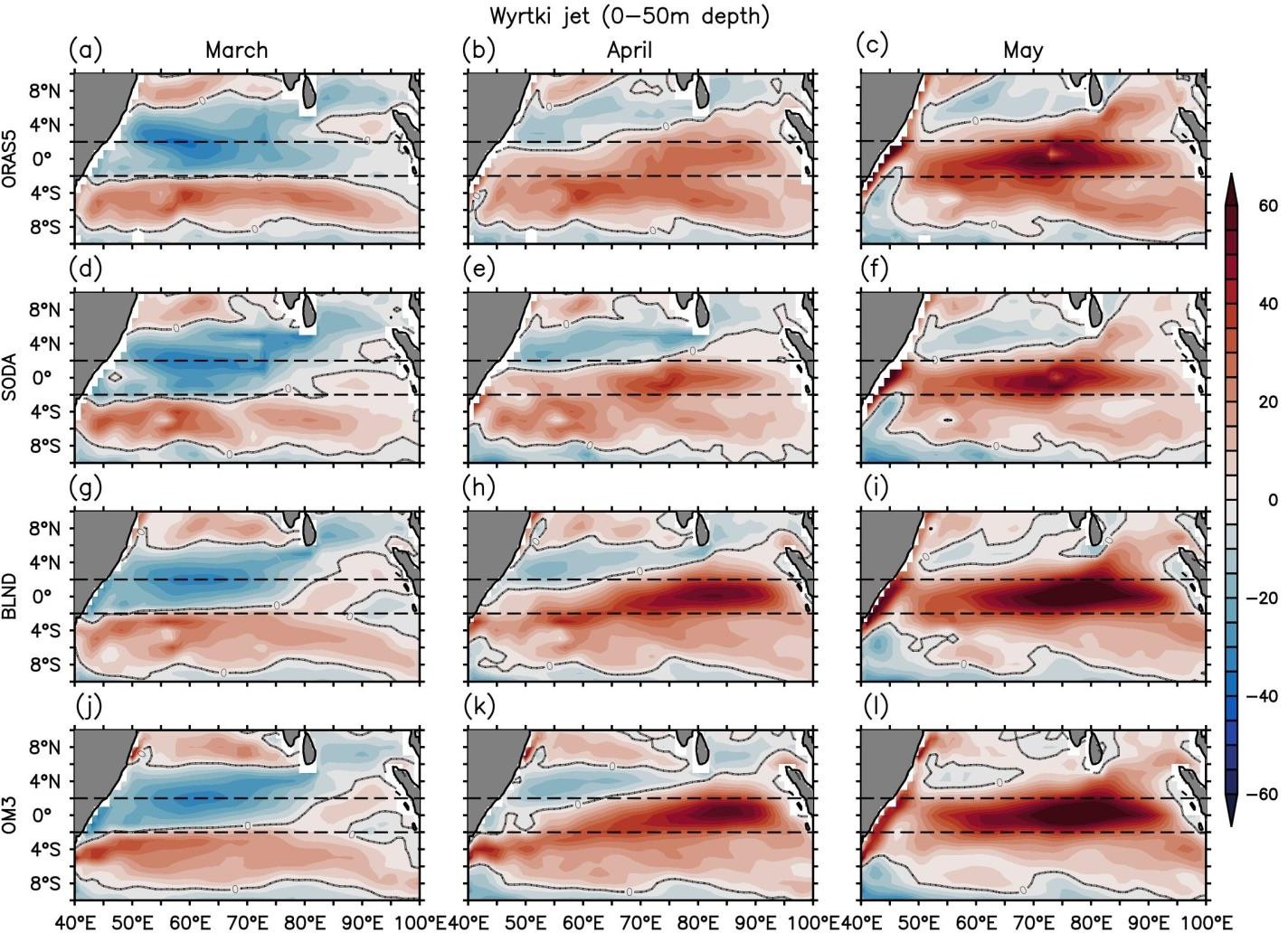
**

**Fig. S1.** Spatial structure of Wyrtki Jet Zonal Current averaged from surface to 50 m depth for the months of March (left panels), April (Middle panels) and May (right panels). The black dashed line shows latitudes 2° N and 2° S.


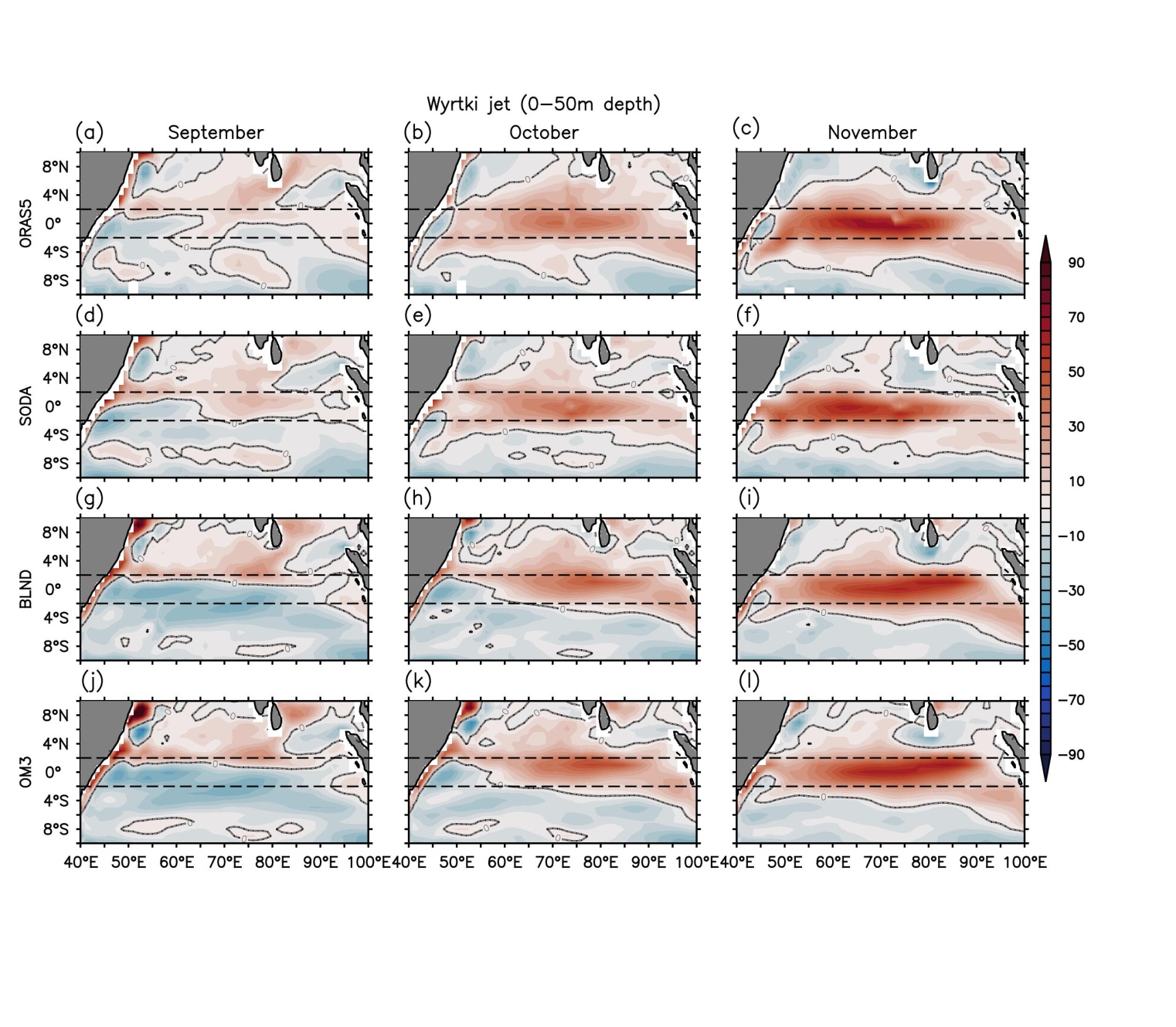


**Fig S2.** Same as Fig. S1 but for the months of September, October, and November.


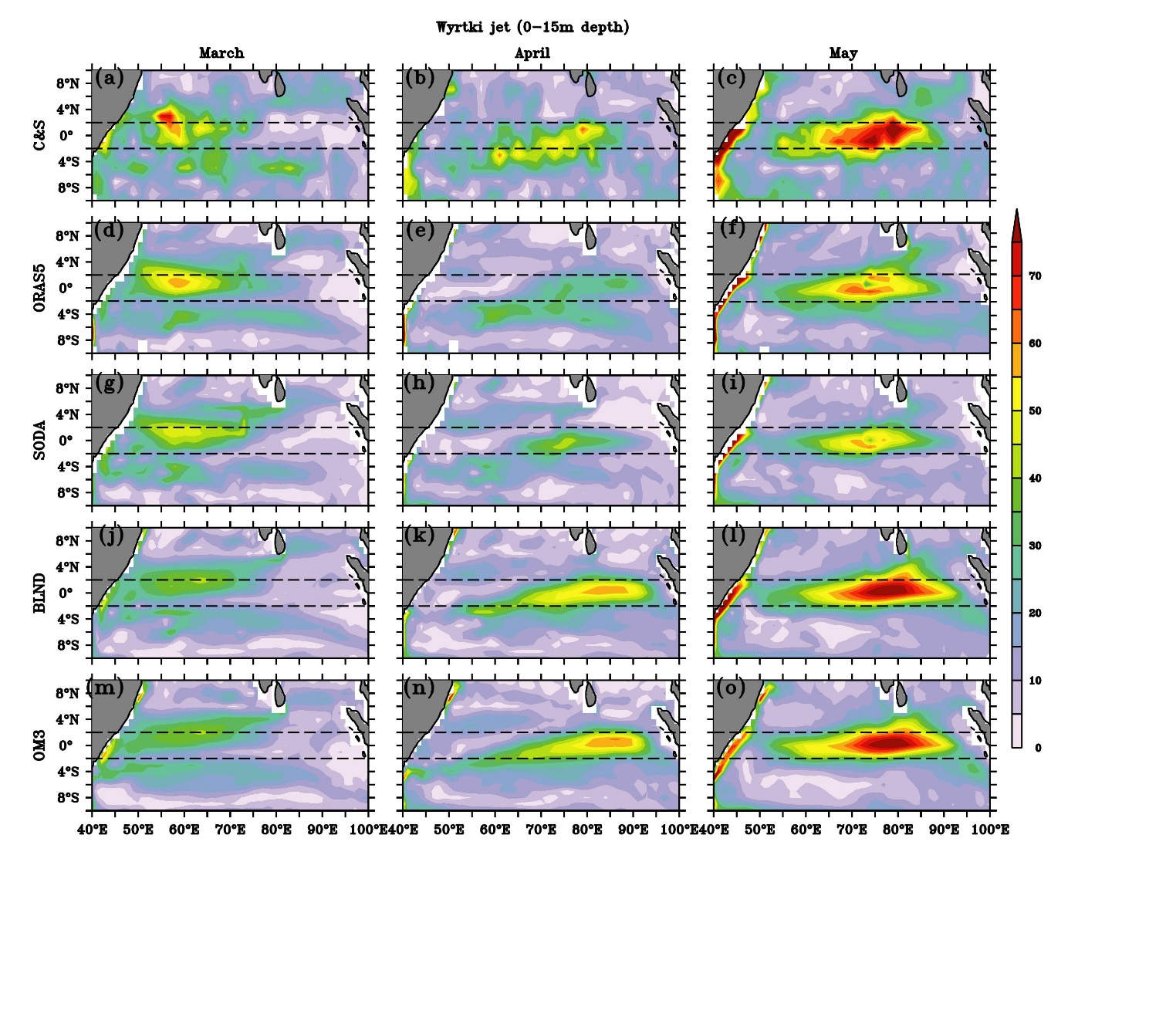


**Fig S3**. The propagation of Wyrtki Jet during spring, March (left panels), April (Middle panels) and May (right panels) averaged for the surface 15 m. (a-c) shows the Cutler and Swallow ship drift currents, (d-f) ORAS5, (g-i) SODA, (j-l) BLND and (m-o) OM3.


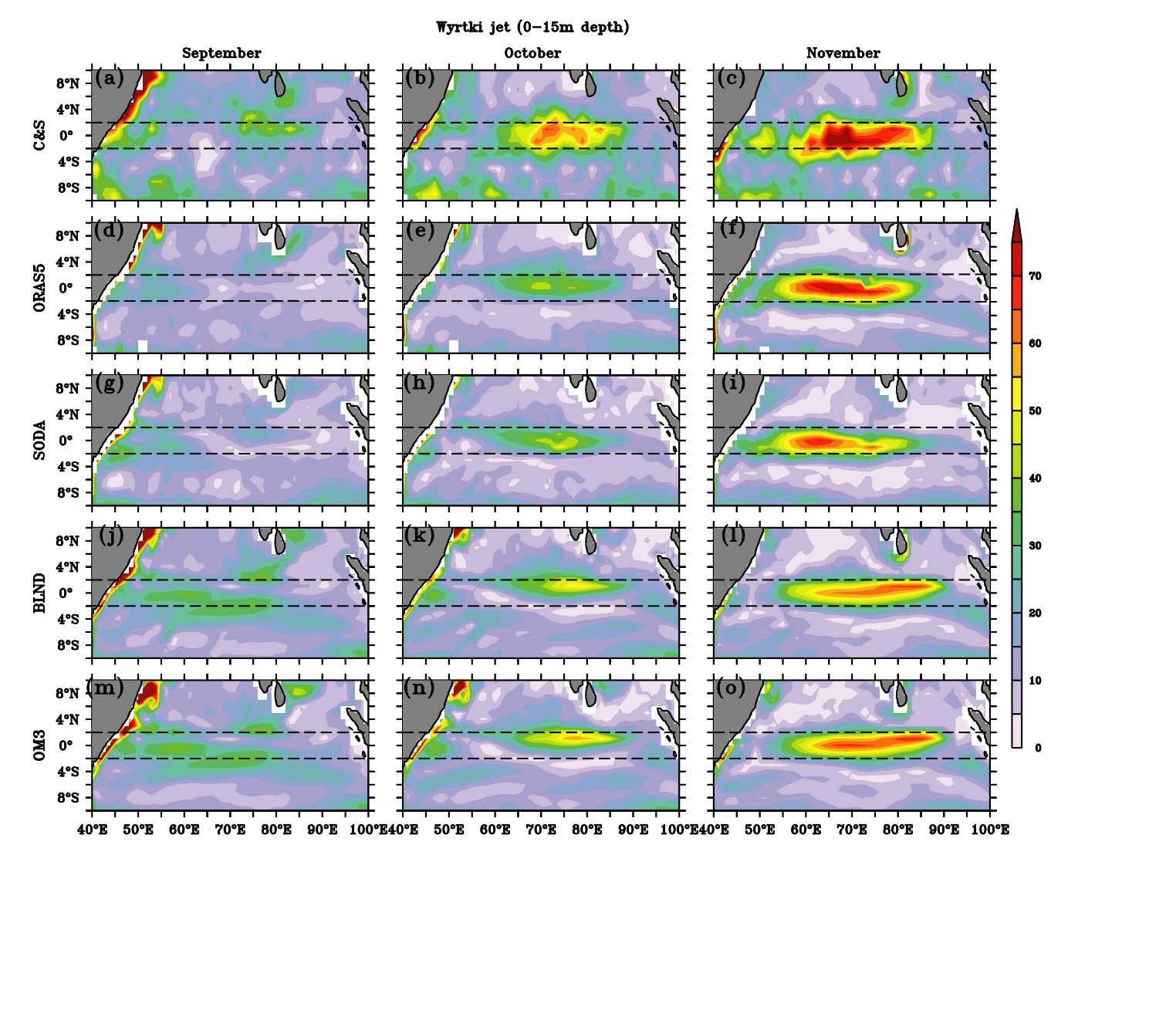


**Fig S4.** Same as S3 but for the months of September (left panels), October (middle panels) and November (right panels).


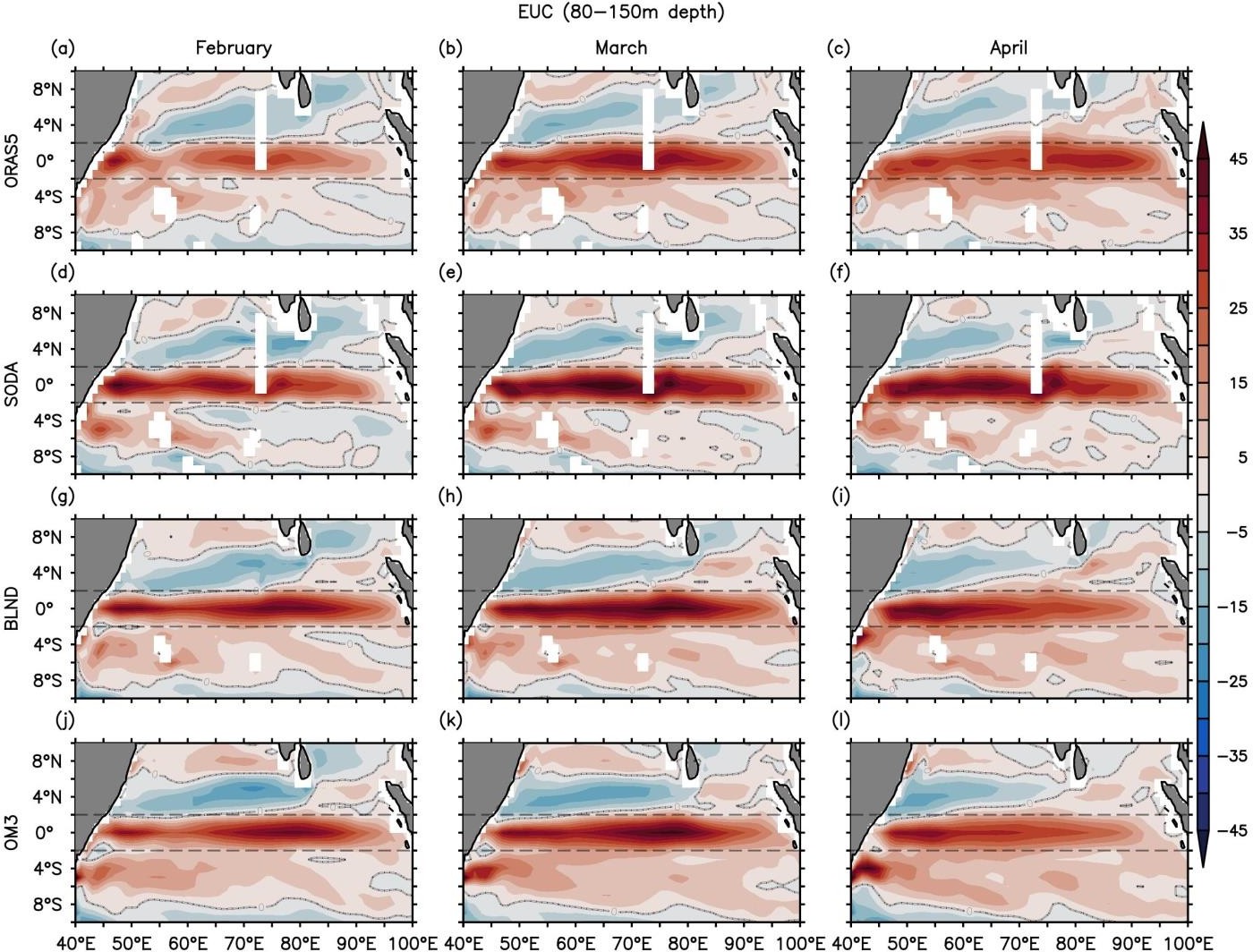


**Fig. S5.** Spatial structure of EUC zonal current for the months of Feb (left panels), Mar (middle panels), and Apr (Right Panels). The currents are averaged from the surface to 80-150 m depth. The black dashed line shows latitudes 2° N and 2° S.


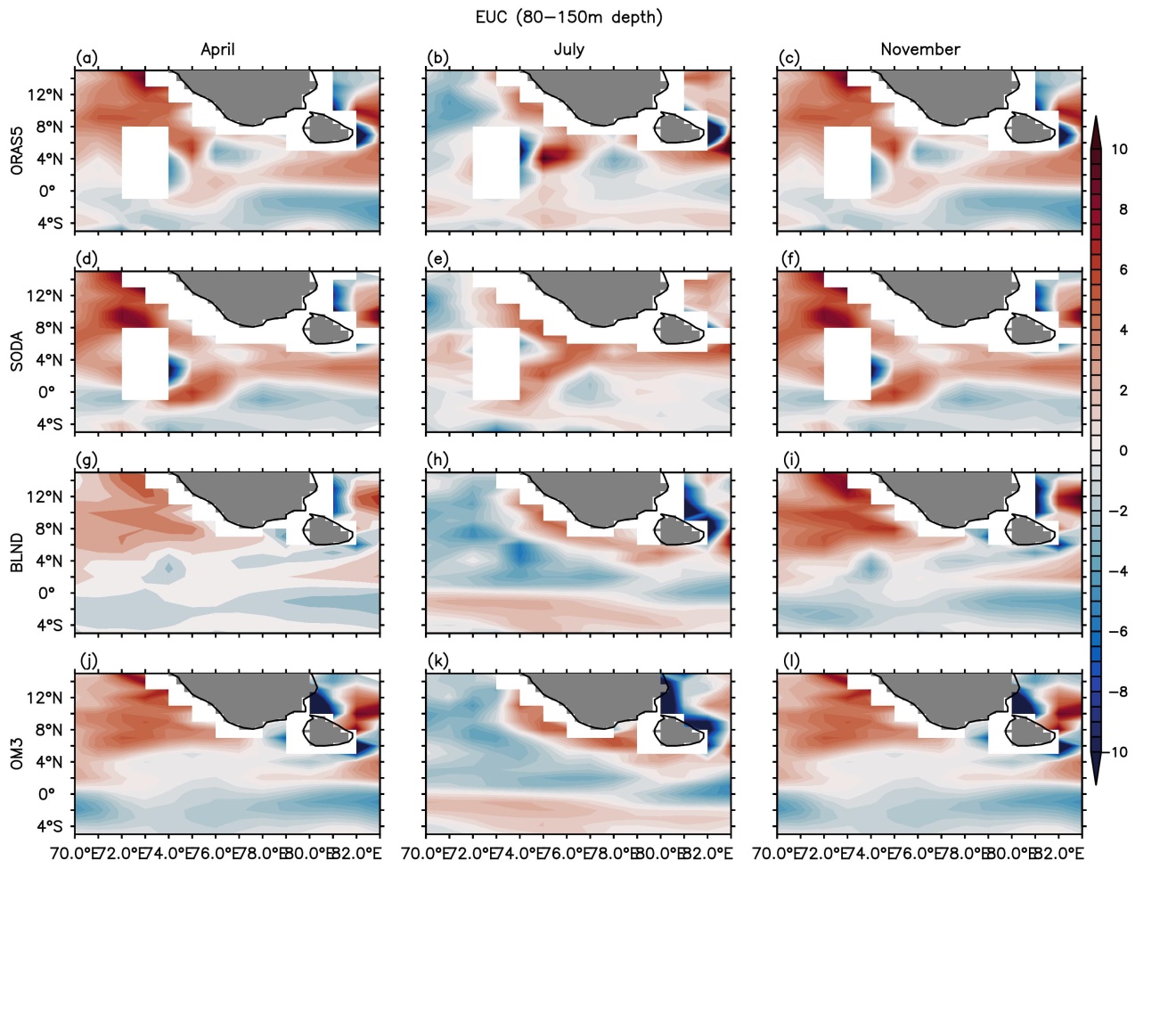


**Fig. S6.** Spatial variations of depth-averaged (80-150 m) Meridional Under current near Maldives.


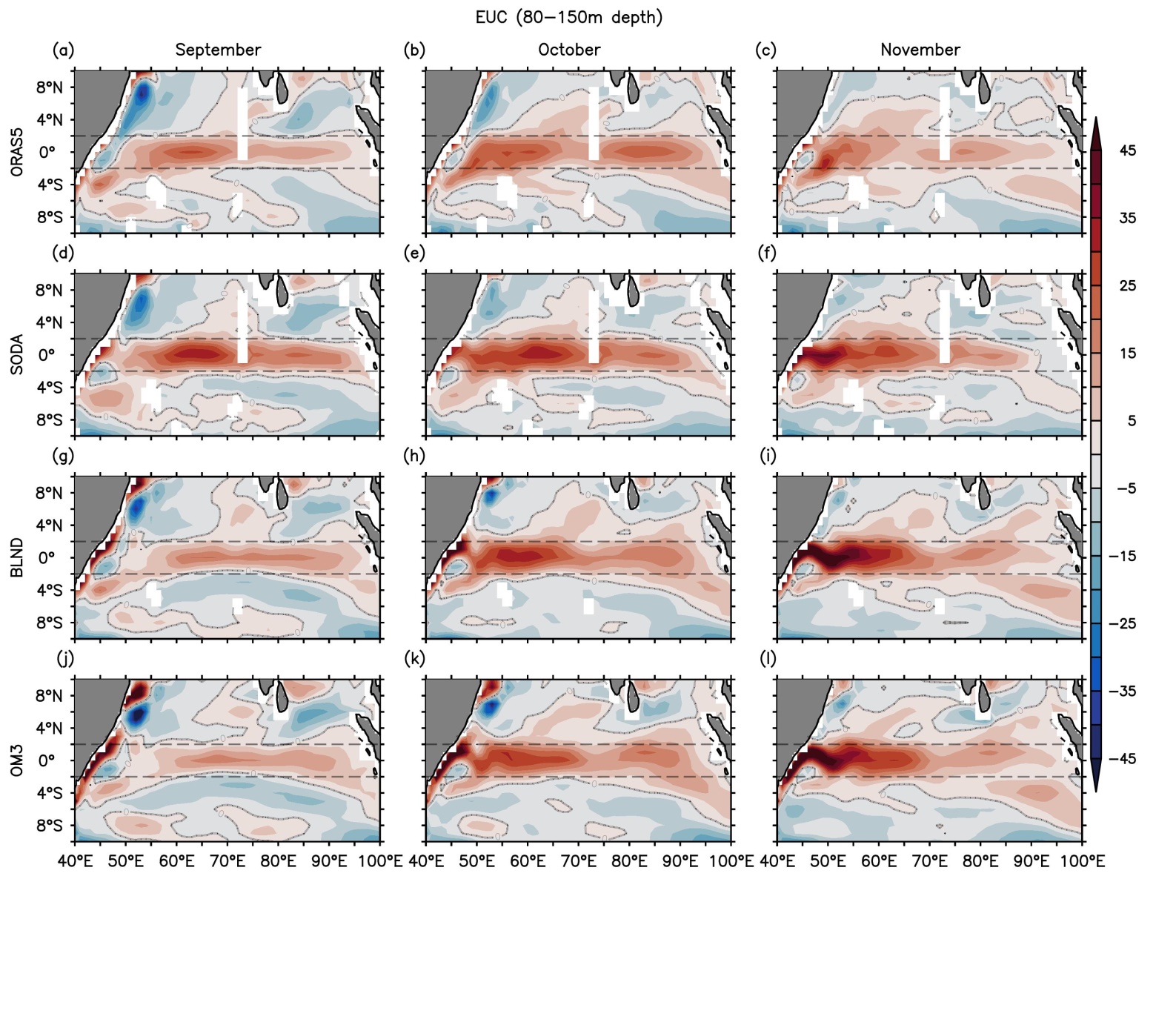


**Fig. S7.** Spatial structure of EUC zonal currents for the months of September (left panels), October (middle panels), and November (Right Panels). The zonal currents are averaged from the surface to 80-150 m depth. The black dashed line shows latitudes 2° N and 2° S.


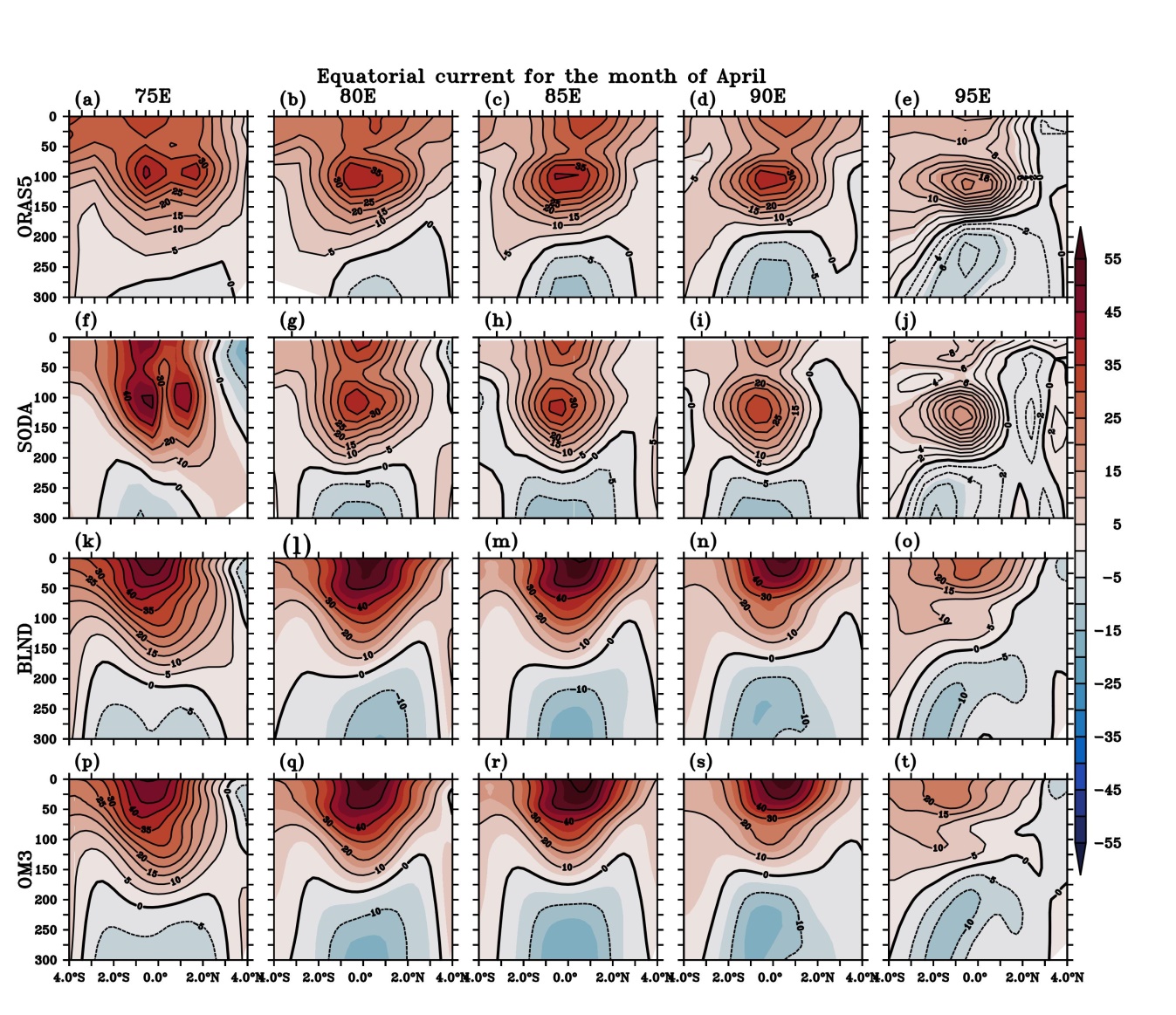


**Fig. S8.** Depth-vs-Latitude sections for 75⁰E, 80⁰E, 85⁰E, 90⁰E and 95⁰E equatorial zonal under current in April.


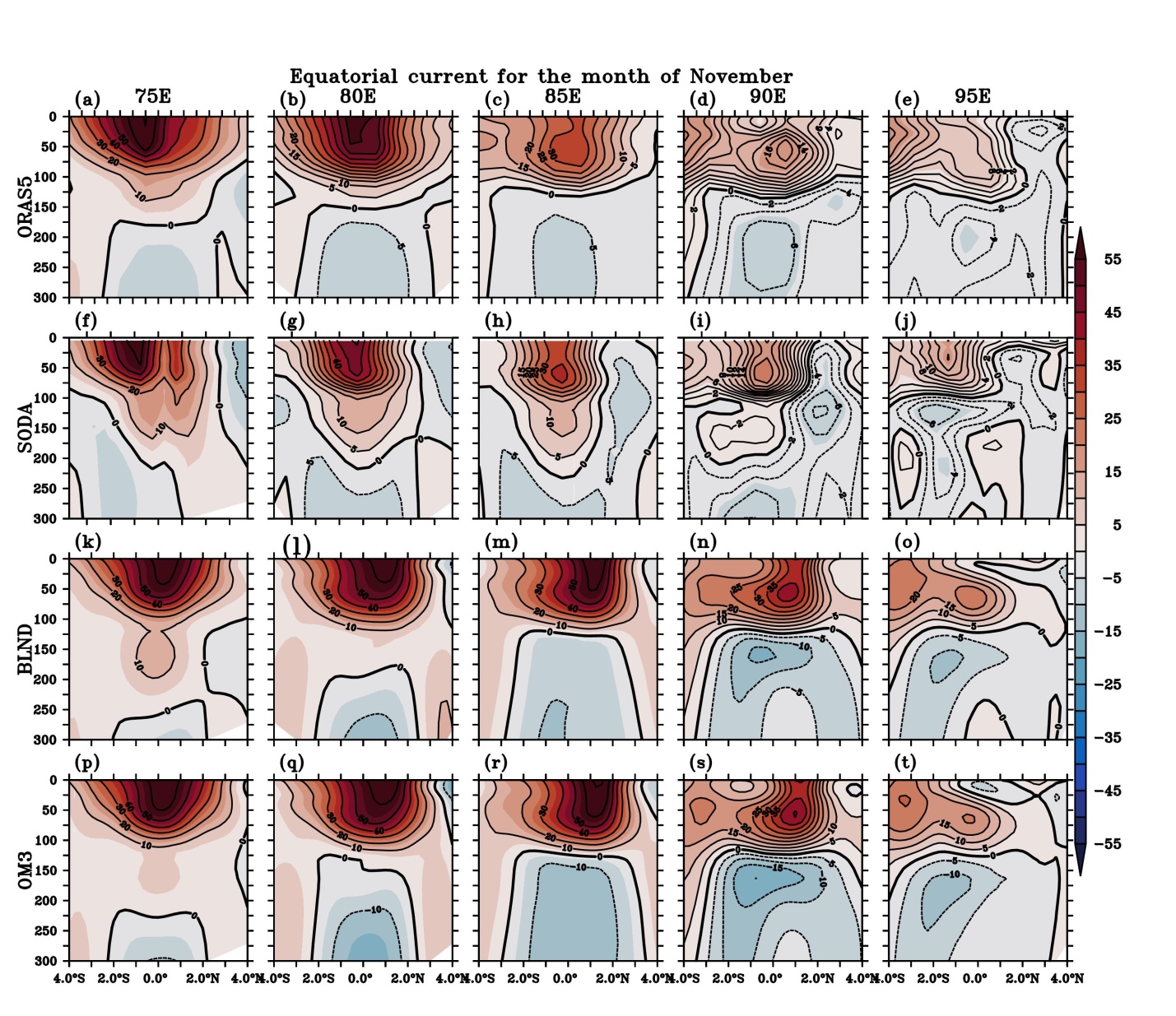


**Fig. S9.** Same as Fig. S8, but for the month of November.

**
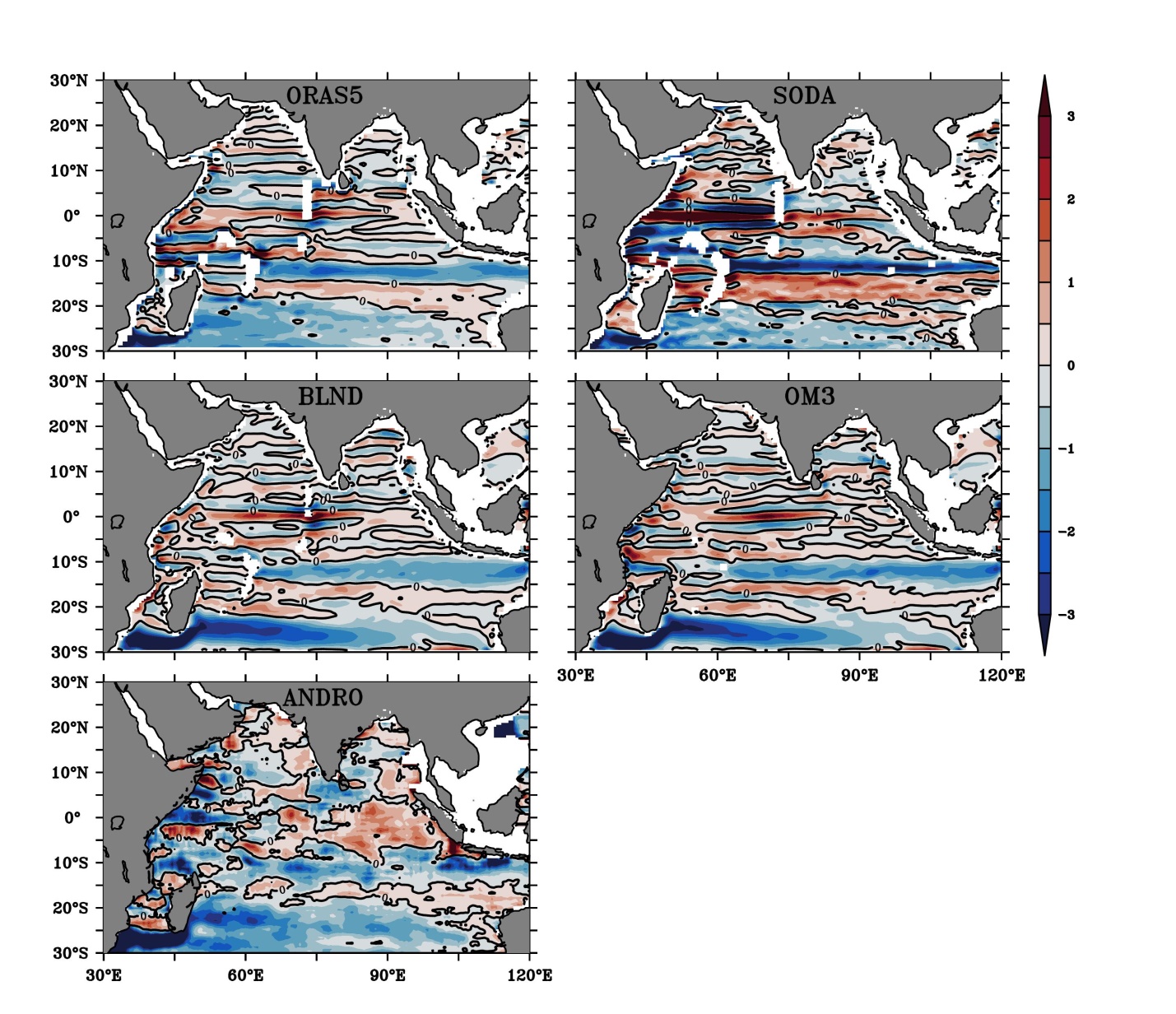
**

**Fig S10.** Spatial distribution of zonal currents at 1000 m depth over the north Indian Ocean domain for ORAS5, SODA, BLND, OM3, and ANDRO currents.

**
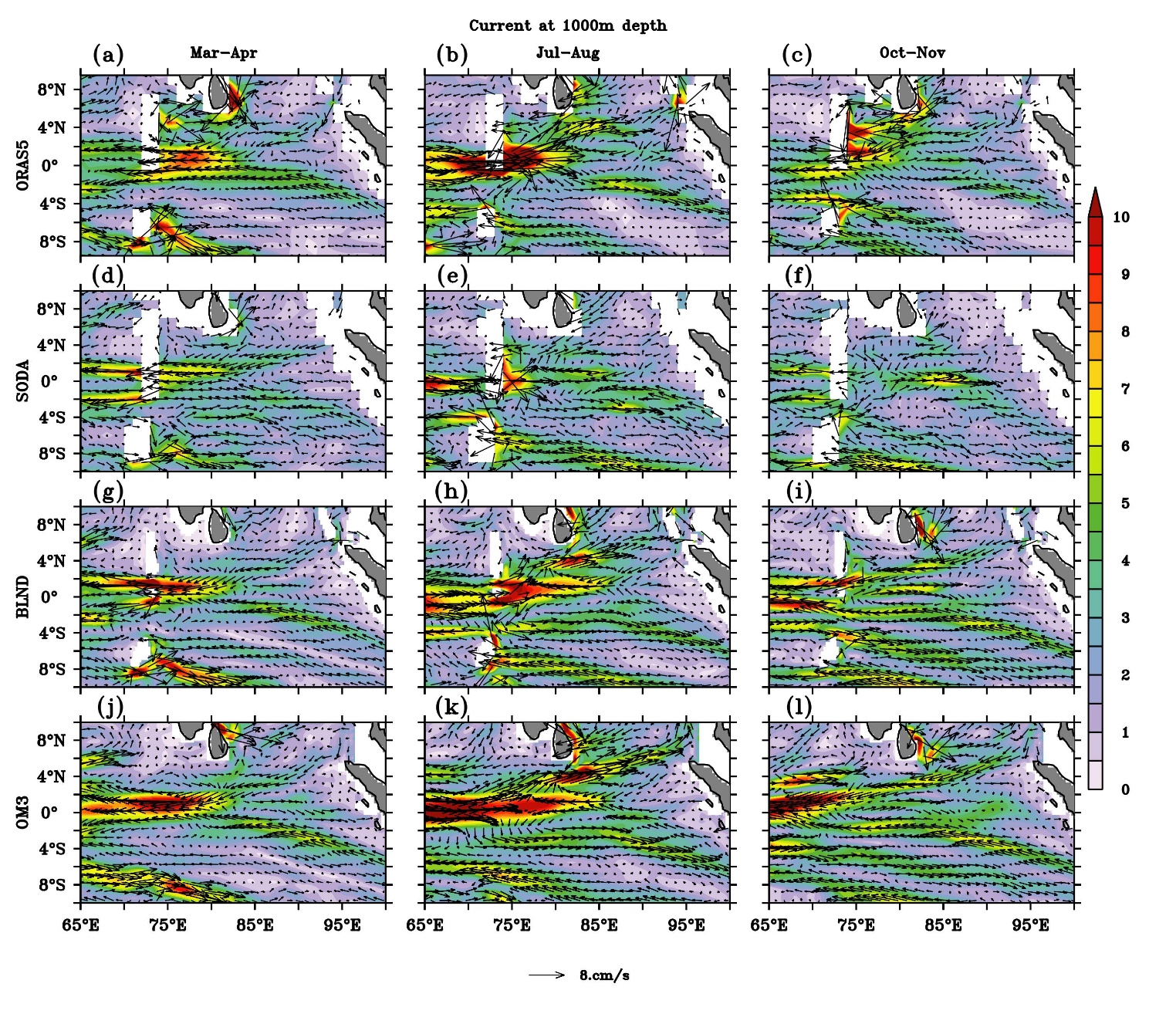
**

**Fig. S11.** Current speed over the central equatorial Indian Ocean at 1000m depth for the months of April (left panels), July (middle panels) and November (Right panels).


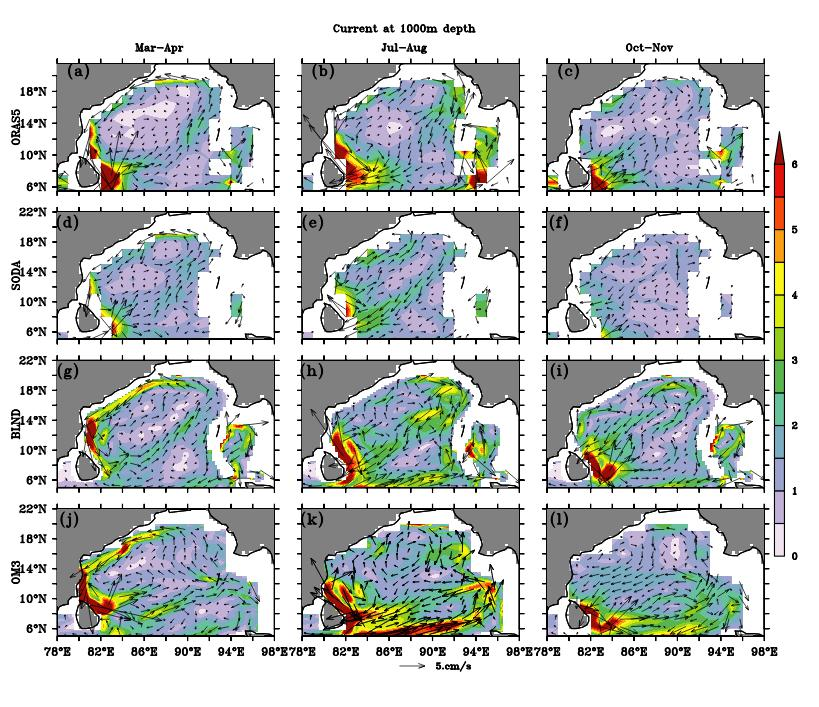


**Fig. S12.** Current speed over the Bay of Bengal at 1000m depth for the months of March-April (left panels), July-August (middle panels) and October-November (Right panels).


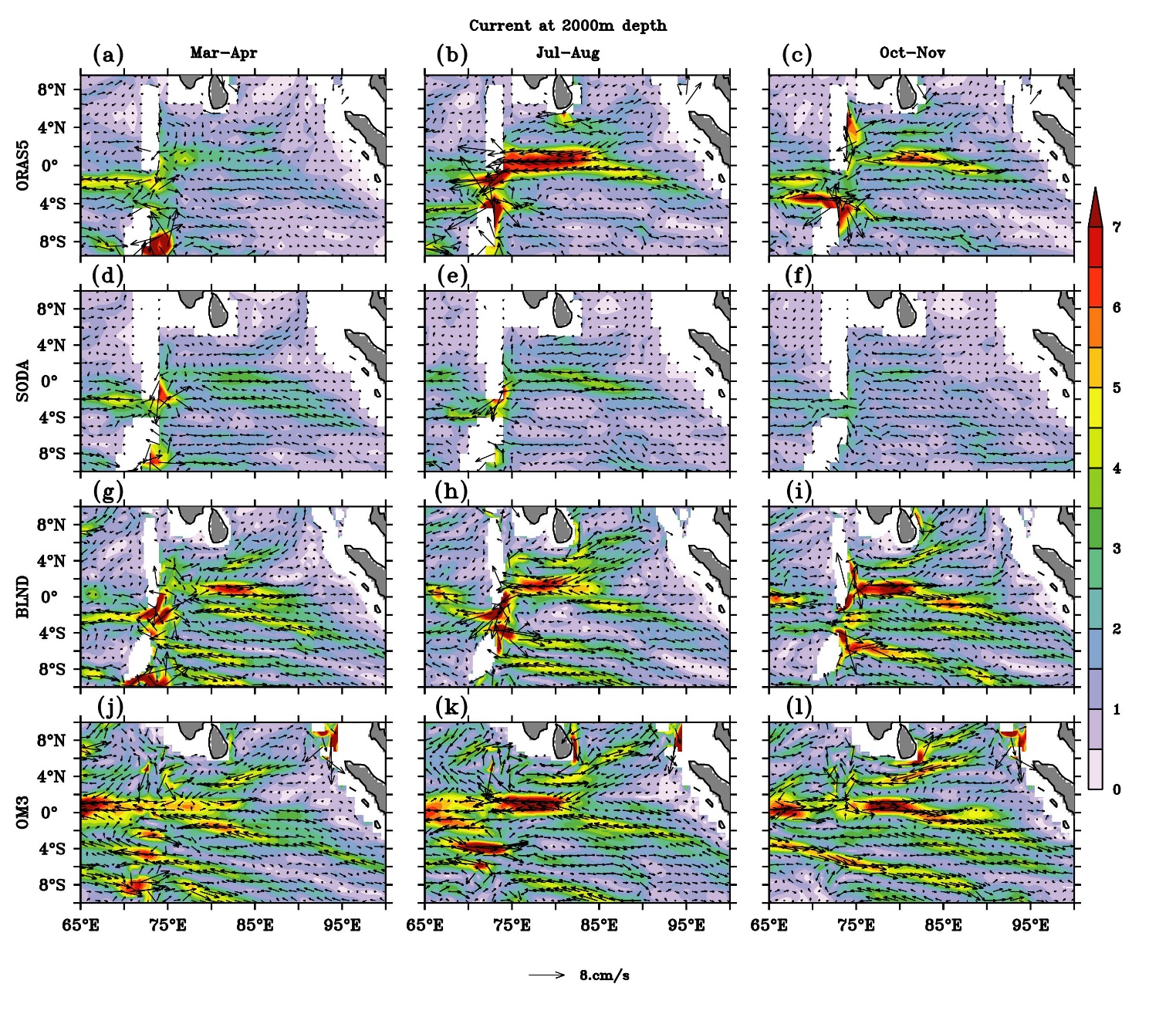


**Fig. S13.** Same as Fig. S11 but for 2000 m depth.


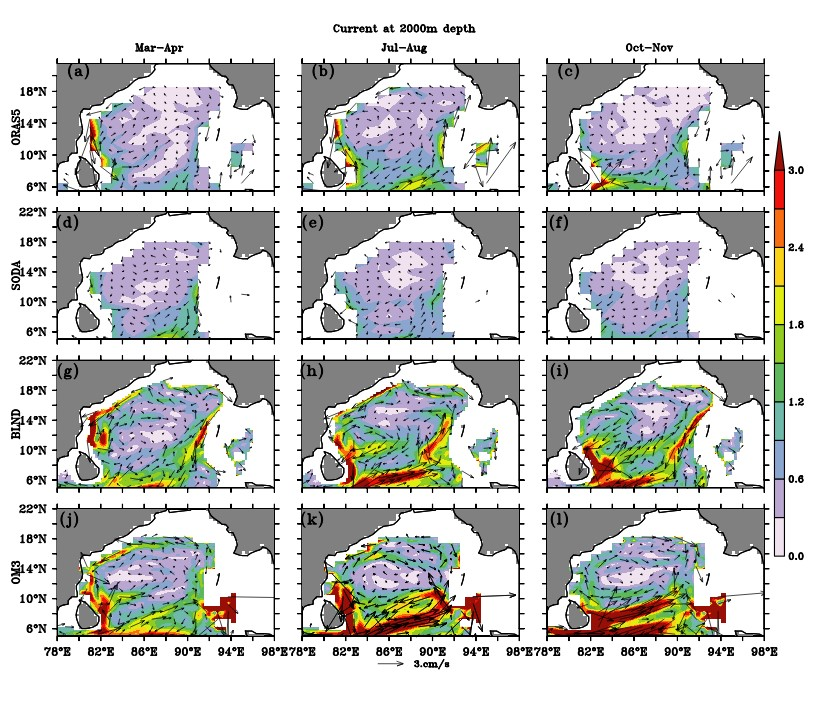


**Fig. S14.** Same as Fig. S12 but for 2000 m depth.
